# Assessment of Strength and Power Capacities in Elite Male Soccer: A Systematic Review of Test Protocols Used in Practice and Research

**DOI:** 10.1007/s40279-024-02071-8

**Published:** 2024-07-18

**Authors:** Nikolaos D. Asimakidis, Irvin N. Mukandi, Marco Beato, Chris Bishop, Anthony N. Turner

**Affiliations:** 1 Faculty of Science and Technology, London Sport Institute, Middlesex University, The Burroughs, London, NW4 4BT UK; 2 Performance Department, Ipswich Town Football Club, Ipswich, UK; 3 School of Health and Sports Sciences, University of Suffolk, Ipswich, UK

## Abstract

**Background:**

Strength and power represent two crucial physical qualities for the attainment of a high level of performance considering the frequency and the importance of explosive actions occurring during elite soccer match-play. Evaluation of strength and power is a multifaceted concept involving a vast array of tests and outcome variables. Nevertheless, a comprehensive and systematic search of strength and power assessment procedures in elite soccer has yet to be undertaken.

**Objectives:**

The aims of this systematic review were to: (1) identify the tests and outcome variables used to assess strength and power of elite male soccer players; (2) provide normative values for the most common tests of strength and power across different playing levels; and (3) report the reliability values of these strength and power tests.

**Methods:**

A systematic review of the academic databases MEDLINE, CINAHL, SPORTDiscus, Web of Science and OVID for studies published until August 2023 was conducted, following the Preferred Reporting Items of Systematic Reviews and Meta-Analyses (PRISMA) guidelines. Studies were eligible for inclusion if they: (1) were original research studies, published in a peer-reviewed journal, and written in English language; (2) had the primary aim to assess strength and/or power; (3) players were male and older than 17 years of age (i.e., mean age of the group); and (4) their playing level was defined as “professional”, “international” or “elite”.

**Results:**

Regarding strength testing, 115 studies and 29 different tests were identified. The three most frequent strength tests were the knee extensor isokinetic strength test (58 studies), the knee flexor isokinetic strength test (55 studies) and the Nordic hamstring strength test (13 studies). In terms of power testing, 127 studies with 31 different tests were included. The three most frequent power tests were the countermovement jump with hands fixed on hips (99 studies), the squat jump (48 studies) and the vertical jump with arm swing (29 studies).

**Conclusions:**

The wide range of different tests and outcome variables identified in this systematic review highlights the large diversity in the employed testing procedures. The establishment of a hybrid testing approach, combining standardised and widely accepted tests for establishing normative standards and enabling comparisons across different contexts, with flexible context-specific testing batteries, has the potential to maximise the impact of testing information for practitioners. In addition, the limited reporting of reliability data across studies highlights the need for practitioners to establish their own reliability measure within their specific contexts, informing the selection of certain tests and outcome variables.

**Supplementary Information:**

The online version contains supplementary material available at 10.1007/s40279-024-02071-8.

## Key Points

**Table:** 

Twenty-nine different strength tests and 31 different power tests were identified in elite soccer.
Isokinetic knee extensor strength, isokinetic knee flexor strength and Nordic hamstring test represent the three most frequent strength tests.
Countermovement jump, squat jump and vertical jump represent the three most frequent power tests.

## Introduction

Soccer is an intermittent sport in which high-to maximum-intensity bouts (i.e. jumping, passing, shooting, tackling, turning, sprinting and changing pace) are interspersed with low-intensity activity [1]. Despite the fact that the explosive actions executed during a soccer match account for only a small percentage of the total distance covered, their role is pivotal given many of them are deemed to be key determinants of success, both at an individual and team level [2]. Specifically, male individuals competing in the top European leagues cover distances of approximately 9–14 km, with approximately 900 m at high speed (> 19.8 km/h), and 300 m at sprinting speed (> 25.2 km/h) [3–5]. In addition, it is common for over 700 changes of direction to be performed in a single match, although some variation exists between playing positions [6]. Furthermore, the physical demands of elite soccer are becoming more demanding, placing increased demands on the players in terms of the quantity and quality of explosive actions [7, 8]. Consequently, possessing a well-developed set of physical attributes such as strength and power is essential for optimising performance and increasing the chances of a long and successful career at the elite level.

Strength and power are key components of an elite soccer player’s physical profile as they largely underpin the successful completion of many of the crucial actions that occur during the game, such as sprinting, jumping, turning, winning physical duels and scoring goals [9–11]. The effectiveness of strength and power interventions in improving the effective execution of various explosive actions such as acceleration, top speed, jumping ability and change of direction has been well documented in elite soccer [12–15]. In addition, previous research has revealed differences in strength and power levels of starting and non-starting, senior and youth, and professional and amateur soccer players [16–18]. Nevertheless, the significance of strength and power is not confined to the concept of performance enhancement. Multiple studies have shown that strength and power can help mitigate against injury [19–22]. Given the fact that elite soccer players are regularly exposed to a congested match and training schedule, maintaining a sufficient level of strength and power is likely to have an influential role in ensuring players are physically robust, thus also reducing the chance of non-contact injuries. Therefore, special consideration needs to be paid to optimising the strength and power outputs of soccer players, which can have a significant impact on both performance and availability to train and compete.

With this in mind, fitness testing constitutes an integral component of the physical development process, as it facilitates the objective assessment of individual and team fitness levels, the comparison of athlete’s performance to normative data, the identification of strengths and weaknesses, and the effectiveness of a training intervention [23, 24]. This can inform decision making on whether to continue or modify a training programme, helping to promote an individualised approach to training prescription [25]. A recently performed survey examining the practices of strength and conditioning coaches in professional soccer settings revealed the importance placed by practitioners on strength and power assessments [26]. However, despite the well-established role of using testing to determine the efficacy of a training programme [24, 27, 28], no large-scale scoping or systematic review has been conducted on the most appropriate and reliable strength and power protocols for soccer. This is somewhat surprising given the popularity of soccer and the vast quantity of assessment methods available to practitioners. Such assessment methods include, but are not limited to: isokinetic dynamometry, repetition maximum (RM) back squat, a variety of isometric strength testing protocols, use of barbell velocity for 1RM estimation, eccentric knee flexor strength via the Nordic hamstring exercise and a plethora of different jumping protocols [24, 29]. This lack of uniformity poses a significant challenge to practitioners, as inconsistent test selection and administration do not allow for the establishment of normative standards. While a standardised testing battery could be valuable for benchmarking purposes, its realisation can be difficult owing to practical constraints, such as time scarcity and equipment availability. As such, testing selection and implementation must be tailored to the specific needs and resources of each setting [30]. In addition, the testing selection process should be influenced by the reliability or repeatability of a test [31], as well as its sensitivity, which refers to the ability of a test to detect small but important changes in performance [32]. If a test cannot be reliably reproduced, practitioners cannot be confident that the test score is an accurate reflection of an athlete’s ability, and whether any subsequent performance changes are true. Hence, practitioners must have a good understanding of these core concepts.

Although previous work has shed light on strength and power testing in soccer [24, 29], a comprehensive and systematic search of strength and power testing in elite soccer is still missing. A systematic review of the literature could offer valuable insights to practitioners working in elite soccer on testing selection, by providing a clear and comprehensive picture of all the available options for strength and power assessments. Furthermore, a potential investigation of the reliability and sensitivity of these tests can support evidence-informed decisions on the strength and power assessments to be used. In addition, as one of the main responsibilities of soccer practitioners is to prepare physically robust athletes that can withstand the demands of the contemporary game, reporting and summarising normative data from studies performed in elite soccer settings could further facilitate the process of strength and power benchmarking. With this in mind, the aims of this systematic review were to: (1) identify the tests and outcome variables used to assess strength and power of elite male soccer players; (2) provide normative values for the most common tests of strength and power across different playing levels; and (3) report the reliability values of these strength and power tests.

## Methods

### Design and Search Strategy

A systematic review was conducted in accordance with the Preferred Reporting Items of Systematic Reviews and Meta-Analyses (PRISMA) statement [33]. A search of the academic databases MEDLINE, CINAHL, SPORTDiscus, Web of Science and OVID was performed from the earliest record to August 2023 to identify English-language, peer-reviewed, original research studies that evaluated strength and/or power ability in elite male soccer players. Keywords employed for the identification of the studies are shown in Table 1. Search levels 1–5 were all linked by the Boolean operator ‘AND’, whereas search terms within each search level were joined with ‘OR’ or ‘NOT’. All search results were extracted and imported into a reference manager software (RefWorks, ProQuest LLC, Ann Arbor, MI, USA).

**Table 1:** Search strategy terms

Soccer OR football NOT (“American football” OR “Australian football” OR rugby OR “Gaelic football”)	Male OR men	Adult OR senior	Professional OR elite	Fitness testing OR physical characteristics OR testing OR physical performance OR physical qualities OR physical profile OR power OR jump* OR strength

### Study Selection

Following the removal of duplicates, two reviewers (NA and CB) independently screened all titles and abstracts against the inclusion and exclusion criteria of the review. Studies that did not meet the inclusion criteria were removed. Conflicts were resolved through discussion, or via a third reviewer (AT). The full text of the articles that were not excluded during this process were subsequently reviewed for eligibility. Supplementary to the systematic search, the reference lists of the included papers were reviewed for the identification of potentially eligible articles. With regard to the first objective of the review, studies were eligible for inclusion if they: (1) were original research studies, published in a peer-reviewed journal, and written in English language; (2) had the primary aim to assess strength and/or power; (3) players were male and older than 17 years of age (i.e. mean age of the group), in line with a previous systematic review on fitness testing (Altmann et al., 2019) and to minimise any potential influence of maturation; and (4) their playing level was defined as “professional”, “international” or “elite”. In contrast, studies were excluded from the review if they: (1) were narrative or systematic reviews and/or meta-analyses; (2) assessed physical characteristics as a result of other research aims (i.e. fatigue, recovery, nutrition and genome); (3) the sample consisted of different team sports; (4) players were semi-professional; and (5) players were younger than 17 years of age. For the second objective, studies were eligible if they reported the mean result of the tests under consideration and clearly distinguished between different groups (i.e. professional vs amateurs, men vs youth, male vs female). As such, only normative data for elite male soccer players older than 17 years old were recorded. For the third objective, studies were included if they provided information about reliability statistics (i.e. within-day and/or between-day).

### Assessment of Methodological Quality

A modified version of the Downs and Black [34] assessment scale was used to evaluate the methodological quality of included articles. This checklist has been used previously in systematic reviews with similar research aims [35, 36] and can be adapted to the scope and the needs of the systematic review [37]. More specifically, 11 questions (1–4, 6, 7, 10, 11, 16, 18, 20) from the traditional version of the checklist were considered relevant with the specific aims of this systematic review, and therefore used to grade the methodological quality of the included studies (Table S1 of the Electronic Supplementary Material [ESM]). For the purposes of this review, question 4 was directed to whether the testing procedures in each study were clearly described. Each question was scored as either a ‘1’ (yes) or a ‘0’ (no or unable to determine). Scores were summed for each study with a total score of ‘11’ representing the highest possible score.

### Data Extraction

Data were extracted and documented using a Microsoft Excel 365 spreadsheet (Microsoft Corporation, Redmond, WA, USA). Extracted data from each study included research design, publication details (authors and year of publication), sample information (number of participants, age of the sample, playing level), tests performed to evaluate strength and/or power ability, outcome measures derived from each test, as well as normative values for each test including reliability values (i.e. intraclass correlation coefficient [ICC], coefficient of variation [CV], standard error of measurement [SEM], minimal detectable change [MDC], Pearson’s *r* and Cronbach’s alpha [α]), where available. Playing level was classified into two distinct categories: (a) senior professionals (i.e. players that were regular members of the first team of a professional soccer club and/or a national team’s senior squad) and (b) elite youth (i.e. players over 17 years of age who were members of the youth department of a professional soccer club, yet not regular members of the first team, were members of a junior national team squad or defined as “elite” by the authors of the study). This distinction was made to account for physiological and training age differences, which is crucial for contextualisation of normative data and more accurate benchmarking. If more than one group of players were investigated in a study, only the groups with a mean age of 17 years or older were considered. To fulfil the purpose of reporting normative values, the mean of each group (i.e. senior professionals vs elite youth) was recorded. When a study consisted of multiple groups of the same playing level, the average of the mean and the pooled standard deviation were recorded. In intervention studies, the baseline values were recorded to eliminate any intervention bias. When a repeated-measures with no intervention study design was implemented (e.g. during seasonal variation studies), the most recent testing point was recorded (except when the most recent point was after a congested fixture period).

## Results

### Identification and Selection of Articles

**Fig. 1:**
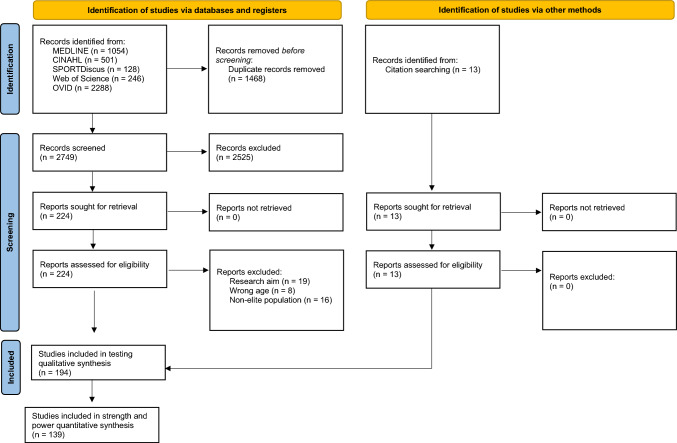
Flow of selection process of eligible studies for a qualitative and quantitative synthesis

The selection process flowchart is presented in Fig. 1. The initial search of databases identified 4217 articles. After removing the duplicates (1468 articles), the titles and the abstracts of 2749 articles were screened. This resulted in the selection of 224 articles to be assessed for eligibility through full-text review. Furthermore, 13 studies were identified through reference lists for full-text eligibility assessment. Following full-text screening, 194 were included for the aim of identifying the tests and outcome variables used to assess strength and power in elite male soccer. Additionally, 139 of these were included for the purpose of reporting normative values for the most common strength and power tests.

### Evaluation of Methodological Quality

Assessment of quality scores can be found in Table S2 of the ESM, with an observed range from 4 to 10 for the 11 items assessed.

### Characteristics of Included Studies

Table S3 of the ESM provides a summary of characteristics of the studies included in this systematic review. The range of sample size was 10–939, with a median of 29 participants. A total of 120 studies included senior professionals as participants, 56 included elite youth, while 18 studies involved a group of both. The age range of the samples involved in the studies was 17.0 to 28.3 years, with a median age of 20.7 years. From a study design standpoint, 100 (52%) studies used a cross-sectional design, 44 (22.9%) were intervention studies, 43 (22.4%) used a repeated-measures design, 5 (2.0%) were reliability studies and 2 (1%) were validity studies. The studies took place in 39 different countries, with Brazil, Spain, Qatar, England and Portugal being the countries with most occurrences.

### Tests and Outcome Variables Used to Assess Strength

Evaluation of strength was performed in 115 studies (59.2%) [Table 2]. A total 29 different tests were used to assess strength, further illustrating the wide range of assessment methods for the evaluation of this physical characteristic. Four different types of strength were evaluated (i.e. isokinetic, isometric, dynamic and eccentric). Isokinetic strength was the most frequently evaluated, being present in 62 studies (54.9%), followed by isometric strength in 29 studies (25.7%), dynamic strength (i.e. the ability to produce force during dynamic movements that include both the eccentric and concentric part, such as squat or bench press) in 27 studies (23.9%), and eccentric strength in 19 studies (15.9%). Isokinetic strength was evaluated by eight different tests, isometric strength by 11 different tests, dynamic strength by seven different tests and eccentric strength by three different tests. The three most frequently occurring tests were: (1) knee extensor isokinetic strength (58 studies); (2) knee flexor isokinetic strength (55 studies); and (3) knee flexor eccentric strength (14 references). It is noteworthy that the hip adductor strength test and the half-back squat test were also frequently employed (12 studies each). Of note, the total number of studies that assessed eccentric hamstring strength with the Nordic hamstring exercise, as well as those that assessed isometric and eccentric strength for the hip adductors and abductors, were all performed during the last decade. Isokinetic dynamometry was the predominant measurement method in the overall number of studies that assessed knee extensor and flexor isokinetic strength. In contrast, Nordic hamstring exercise was the primary measurement method to assess knee flexor eccentric strength (13 studies). For knee isokinetic strength, peak concentric torque (48 studies), conventional strength hamstrings/quadriceps ratio (28 studies) and relative peak concentric torque (17 studies) were the main outcome variables. In terms of knee flexor isokinetic strength, the three main outcome variables were peak concentric torque (46 studies), peak eccentric torque (28 studies) and relative peak concentric torque (16 studies). Last, peak force (13 studies) was the main outcome variable in the assessment of eccentric knee flexor strength via the Nordic hamstring exercise.

**Table 2:** Strength tests and outcome variables

Test	Equipment/method	Outcome variable	References
Knee extensor isokinetic strength	Isokinetic dynamometer	Peak concentric torque (Nm)	[18, 20, 21, 42, 85, 94, 101, 105, 106, 123, 126, 133, 137, 140, 148, 152–155, 170, 173, 177–179, 181, 184, 186, 190, 197, 200, 202, 208, 210, 211, 213, 220, 221, 229, 232, 235, 236, 238, 241, 244, 246, 248, 251, 252]
		Conventional strength hamstrings/quadriceps ratio	[18, 20, 21, 42, 43, 94, 101, 105, 106, 132, 154, 155, 160, 173, 181, 184, 186, 209, 211, 213, 221, 229, 235, 244, 246, 248, 249, 252]
		Relative peak concentric torque (Nm/kg)	[21, 42, 43, 66, 94, 132, 142, 149, 155, 160, 170, 179, 201, 210, 220, 221, 232]
		Functional strength hamstrings/quadriceps ratio	[18, 20, 42, 105, 106, 126, 133, 155, 173, 177, 178, 184, 186, 209, 246, 248]
		Peak eccentric torque (Nm)	[106, 123, 173, 177, 184, 188, 208, 244, 246, 248]
		Relative peak eccentric torque (Nm/kg)	[142, 149]
		Mean power (W)	[154, 232]
		Relative total work (J/kg)	[132, 160]
		Relative total work (Nm/kg)	[201]
		Total work (J)	[152]
		Relative mean power (W)	[232]
		Functional range (°)	[133]
Knee flexor isokinetic strength	Isokinetic dynamometer	Peak concentric torque (Nm)	[18, 20, 21, 42, 94, 101, 105, 106, 123, 126, 137, 140, 148, 152–156, 170, 173, 177, 178, 181, 184, 186, 190, 197, 200, 208, 210, 211, 213, 220, 221, 229, 232, 235, 238, 240, 241, 244, 246, 248, 249, 251, 252]
		Peak eccentric torque (Nm)	[18, 20, 21, 42, 101, 105, 106, 123, 126, 133, 140, 153, 155, 156, 170, 173, 177, 178, 184, 186, 188, 190, 208, 210, 240, 244, 246, 248]
		Relative peak concentric torque (Nm/kg)	[21, 42, 43, 66, 94, 132, 142, 149, 155, 160, 170, 202, 210, 220, 221, 232]
		Relative peak eccentric torque (Nm/kg))	[21, 42, 142, 149, 170, 210]
		Relative total work (J/kg)	[132, 160]
		Mean power (W)	[154, 232]
		Total work (J)	[152]
		Relative mean power (W)	[232]
		Functional range (°)	[133]
Knee flexor eccentric strength	Nordic	Peak force (N)	[42, 45, 95, 104, 107, 121, 122, 126, 131, 153, 253]
		Relative peak force (N/kg)	[42, 45, 104, 131]
		Between-limb mean peak force (N)	[121]
		Mean eccentric torque	[87]
		Break-point angle (°)	[156]
	Hand-held dynamometer	Strength (kg)	[210]
		Relative strength (kg)	[210]
Hip adductor isometric strength	Hand-held dynamometer	Relative peak force (N/kg)	[141, 142, 146, 165]
		Relative peak torque (Nm/kg)	[50, 108, 163]
		Peak force (N)	[141, 146, 155]
		Adduction/abduction ratio	[50, 108, 116]
		Peak torque (Nm)	[116]
		Mean force (N)	[116]
	ForceFrame	Peak force (N)	[114, 124]
		Peak torque (Nm)	[114]
		Relative peak torque (Nm/kg)	[114]
		Relative peak force (N)	[114]
	GroinBar	Mean isometric force (N)	[87]
Half-back squat	Barbell	1RM (kg)	[9, 80, 119, 135, 177, 215, 216, 222, 231, 242]
		Relative 1RM (1RM/kg)	[135]
	Smith machine	1RM (kg)	[159, 217]
Knee extensor isometric strength	Isokinetic dynamometer	Peak torque (Nm)	[85, 105, 135, 219, 238]
		Conventional rate of torque development hamstrings/quadriceps ratio	[105, 219]
		Peak rate of torque development (Nm/s)	[85, 105]
		Relative peak torque (Nm/kg)	[135],
		Peak force (N)	[177]
		Conventional strength hamstrings/quadriceps ratio	[219]
		Rate of torque development 50 ms (Nm/s)	[85]
		Rate of torque development 150 ms (Nm/s)	[85]
		Rate of torque development 250 ms (Nm/s)	[85]
	Hand-held dynamometer	Relative peak torque (Nm/kg)	[99, 111]
		Peak force (N)	[111]
		Peak torque (Nm)	[111]
		Hamstrings/quadriceps ratio	[111]
		Strength (kg)	[210]
		Relative strength (kg)	[210]
	Torque meter	Peak torque (Nm)	[154]
Back squat	Barbell	1RM (kg)	[14, 15, 100, 203, 247]
		Relative 1RM (1RM/kg)	[15]
	Smith machine	1RM (kg)	[16, 172]
		Relative 1RM (1RM/kg)	[16]
	Keiser squat air pneumatic machine	1RM (kg)	[129]
Hip abductor isometric strength	Hand-held dynamometer	Relative peak torque (Nm/kg)	[50, 99, 108, 116]
		Relative peak force (N/kg)	[112, 142]
		Peak torque (Nm)	[116]
		Peak force (N)	[112]
		Mean force (N)	[116]
	GroinBar	Mean isometric force (N)	[87]
	ForceFrame	Peak force (N)	[124]
Knee flexor isometric strength	Hand-held dynamometer	Relative peak force (N/kg)	[90, 104]
		Peak force (N)	[104, 111]
		Relative peak torque (Nm/kg)	[99, 111]
		Peak torque (Nm)	[111]
		Strength (kg)	[210]
		Relative strength (kg)	[210]
	Isokinetic dynamometer	Peak torque (Nm)	[105, 211]
		Rate of torque development (Nm/s)	[105, 211]
Hip adductor eccentric strength	Hand-held dynamometer	Relative torque (Nm/kg)	[50, 145, 146, 165]
		Peak force (N)	[126, 155]
		Adduction/abduction ratio	[126, 155, 165]
Bench press	Barbell	1RM (kg)	[17, 100, 222, 242, 247]
Hip abductor eccentric strength	Hand-held dynamometer	Relative torque (Nm/kg)	[145, 146, 165]
		Peak force (N)	[126, 155]
Isoinertial squat-loading test	Smith machine	Average mean concentric velocity (m/s)	[223]
		Average mean propulsive velocity (m/s)	[166]
		Estimated 1RM (kg)	[166]
		Relative load that elicited mean propulsive velocity of 1 m/s (kg/BM)	[180]
Leg press test	Keiser	Peak power (W)	[86, 92]
		Relative peak power (W/kg)	[86]
		Peak force (N)	[86, 189]
		Relative peak force (N/kg)	[86]
	Leg press	1RM (kg)	[183]
Hip extensor isometric strength	Hand-held dynamometer	Relative peak torque (Nm/kg)	[99, 125]
		Relative peak force (N/kg)	[90]
Ankle plantarflexion isokinetic strength	Isokinetic dynamometer	Peak concentric torque (Nm)	[102, 251]
		Relative peak concentric torque (Nm)	[102]
		Total work (J)	[102]
		Average power (W)	[102]
		Plantarflexion/dorsiflexion ratio (%)	[102]
Ankle dorsiflexion isokinetic strength	Isokinetic dynamometer	Peak concentric torque (Nm)	[102, 251]
		Relative peak concentric torque (Nm)	[102]
		Total work (J)	[102]
		Average power (W)	[102]
Isometric midthigh pull	Force plates	Peak force (N)	[59]
		Relative peak force (N/kg)	[59]
		Peak rate of force development (N/s)	[59]
		Force at 100 ms (N)	[59]
		Relative force at 100 ms (N)	[59]
Hip external rotator isometric strength	Hand-held dynamometer	Relative peak torque (Nm/kg)	[99, 125]
Hip flexor isometric strength	Hand-held dynamometer	Relative peak torque (Nm/kg)	[50]
Modified Thomas test (hip flexor isometric strength)	Hand-held dynamometer	Relative peak torque (Nm/kg)	[50]
Hip flexor isokinetic strength	Isokinetic dynamometer	Peak concentric torque (Nm)	[251]
Hip extensor isokinetic strength	Isokinetic dynamometer	Peak concentric torque (Nm)	[251]
Hip adductor isokinetic strength	Isokinetic dynamometer	Peak concentric torque (Nm)	[169, 251]
		Adductor/abductor ratio (%)	[169]
Hip abductor isokinetic strength	Isokinetic dynamometer	Peak concentric torque (Nm)	[169, 251]
Isometric clam test	Fixed dynamometer	Peak force (N)	[112]
		Relative peak force (N/kg)	[112]
Core endurance test		Time to exhaustion (s)	[17, 127]
Rear foot elevated split squat test	Barbell	1RM (kg)	[14]
Abdominal isometric strength test	ABTest system, isometric device	Strength (kg)	[137]
		Power	[137]
		Fatigue index	[137]

*1RM* one repetition maximum

### Tests and Outcome Variables used to Assess Power

Evaluation of power was performed in 127 studies (65.4%) [Table 3]. Thirty-one different tests were used to assess power in elite soccer players, employing primarily various types of jumps (24 different in total). The countermovement jump (CMJ) with hands fixed on hips (99 studies), squat jump (SJ) (48 studies) and vertical jump with the use of an arm swing (VJ) [29 studies] were the most frequently utilised. Among these, jump height was by far the most common outcome variable reported in 95 studies in the CMJ, 47 studies in the SJ and 27 studies in the VJ. However, it is important to note that the calculation of jump height was based on different methods (e.g. impulse-momentum method, flight time method) owing to the different equipment used. Furthermore, two additional commonly reported outcome variables in CMJ were relative peak power (W/kg) [nine studies] and peak power (W) [five studies]. Among unilateral tests, the single-leg CMJ test (SLCMJ) was the most frequently implemented, featuring in 12 studies. It is noteworthy that all of those studies were performed during the last decade. Finally, the drop jump test (used to assess reactive strength ability) [38] was reported in eight studies.

**Table 3:** Power tests and outcome variables

Test	Outcome variable	References
CMJ (hands on hips)	Height (cm)	[12–14, 16, 19, 59, 62–66, 75, 80, 84, 86–88, 91, 93, 94, 96–98, 100, 101, 103, 109, 115, 117, 119, 120, 126, 128, 130, 131, 134, 138, 139, 143, 144, 147, 149, 151, 152, 154, 157–159, 161, 164, 166–168, 171, 174, 175, 177, 180, 182, 183, 185, 187, 188, 191, 192, 194–196, 199, 200, 202–205, 207, 212–216, 218, 221, 223–228, 230, 231, 236, 237, 239, 241, 243, 244]
	Relative peak power (W/kg)	[13, 59, 66, 86, 113, 154, 158, 201, 239]
	Peak power (W)	[59, 91, 154, 208, 239]
	Peak concentric force (N)	[91, 120, 126, 185, 239]
	RSI-mod	[64, 66]
	Relative peak concentric force (N/kg)	[113, 239]
	Time to take-off (ms)	[64]
	Concentric impulse (Ns)	[120]
	Countermovement depth (cm)	[64]
	Relative net impulse (Ns/kg)	[201]
	Fmax (BW)	[201]
	Eccentric leg stiffness (N/m)	[113]
	Rate of force development (N/s)	[120]
SJ	Height (cm)	[15–17, 19, 21, 65, 75, 80, 84, 87, 88, 96, 97, 100, 103, 109, 119, 127, 128, 131, 138, 139, 144, 149, 151, 152, 164, 175, 177, 195, 196, 200, 203–205, 207, 212, 214, 216, 221, 227, 228, 231, 232, 234, 241, 243, 244]
	Peak velocity at take-off (m/s)	[232]
	Eccentric utilisation ratio	[84]
	Peak power (W)	[232]
Vertical jump/CMJ (free arms)	Height (cm)	[9, 17, 110, 115, 118, 127, 134, 135, 154, 161, 162, 168, 171, 174, 176, 187, 192, 205, 206, 225, 226, 232, 233, 238, 242, 243, 247]
	Peak power (W)	[135, 154, 232]
	Velocity at take-off (m/s)	[198, 232]
	Relative peak power (W/kg)	[135, 154]
	Flight time (s)	[198]
	Predicted power (W)	[233]
Unilateral CMJ	Height (cm)	[14, 59, 63, 64, 66, 77, 84, 126, 154, 158, 187, 194]
	Relative peak power (W/kg)	[66, 154, 158]
	RSI-mod	[64, 66]
	Peak power (W)	[154]
	Concentric impulse (Ns)	[77]
	Time to take-off (ms)	[64]
	Countermovement depth (cm)	[64]
	Concentric peak force (N)	[126]
Drop jump	Height (cm)	[59, 96, 175, 182, 218, 233]
	Contact time (ms)	[59, 182, 198, 218]
	RSI	[14, 59, 182]
	Flight time (s)	[198]
	Stiffness	[59]
	Velocity at take-off (m/s)	[198]
	Predicted power (W)	[233]
Jump squat	Relative peak power (W/kg)	[75, 130, 139]
	Peak power (W)	[231]
	Relative mean concentric power	[139]
	Mean propulsive velocity (m/s)	[183]
	Mean power (W)	[151]
	Mean propulsive power (W)	[151, 203]
	Relative mean propulsive power (W/kg)	[139, 144, 175]
Half-squat	Mean propulsive power (W)	[150]
	Peak power (W)	[194]
	Relative mean propulsive power (W/kg)	[75, 97, 144]
Unilateral drop jump	Height (cm)	[59, 77, 84]
	RSI	[14, 59, 77]
	Contact time (ms)	[59]
Standing broad jump	Distance (m)	[14, 110, 136, 185]
	Peak force (N)	[185]
Wingate anaerobic test (10 s)	Peak power (W)	[93, 135, 245]
	Relative peak power output (W/kg)	[93, 135]
	Mean power for the first 5 s	[245]
	Mean power for the first 10 s	[245]
Five-jump test	Distance (m)	[193, 208, 232]
Single-leg hop test	Distance (cm)	[14, 250]
	Relative peak power (W/kg)	[158]
CMJ (with external load on Smith machine)	Height (cm)	[223]
CMJs for 15 s	Mean height (cm)	[225]
	Relative peak power (W/kg)	[243]
CMJ (pole on shoulders)	Height (cm)	[172]
Unilateral 10-s hop test	Height (cm)	[126]
	RSI	[126]
Keiser squat air pneumatic machine at 50% 1RM	Peak power (W)	[129]
Unilateral half-squat	Mean propulsive power (W)	[150]
Repeated vertical jump test (15 maximum continuous vertical jumps)	Best height (cm)	[89]
	Mean height (cm)	[89]
	Best RSI	[89]
	Mean RSI	[89]
	Relative peak power (W/kg)	[89]
30-s jumping test (CMJs for 30 s)	Mean height (cm)	[152]
3–4 step run-up jump	Height (cm)	[154]
	Peak power (W)	[154]
	Relative peak power (W/kg)	[154]
Soccer-specific vertical jump	Flight time (s)	[198]
	Velocity at take-off (m/s)	[198]
60% 1RM squat	Mean power (W)	[203]
Continuous jumps with leg extended	Jump height (cm)	[214]
CMJs for 20 s	Mean height (cm)	[230]
4-bounce test (4BT)	Distance (m)	[231]
Triple-hop test	Distance (m)	[136]
Squat (Smith machine)	Mean power (W)	[19]
Maximal effort standing cycle test (10 s)	Alactic power index (W/kg)	[249]
Single-leg medial hop test	Relative peak power (W/kg)	[158]
Timed single-leg hop for 6 m	Time (s)	[250]

*1RM* one repetition maximum, *CMJ* countermovement jump, *Fmax* maximum force, *RSI* reactive strength index, *RSI-mod* reactive strength index modified, *SJ* squat jump

### Reliability Data

Reliability statistics reported for the strength and power tests can be found in Tables S4 and S5 of the ESM, respectively. For strength tests, reliability statistics were reported in 15 studies (13%). Intra-day reliability was the most common reliability type reported in nine studies, while inter-day reliability was determined in five studies. One study also assessed inter-season reliability. In terms of specific reliability metrics, the reported metrics were ICC (13 studies), SEM (seven studies), CV (four studies), MDC (two studies) and Cronbach’s alpha (one study). Knee extensor isokinetic strength (four studies), knee flexor isokinetic strength (four studies), half-back squat (four studies) and Nordic hamstring testing (three studies) were the tests for which reliability values were most reported. Intra-day reliability values (i.e. ICC, CV, SEM) were higher compared to inter-day and inter-season reliability for all these tests. In terms of power tests, reliability values were reported in 34 studies (27%). Intra-day reliability was the most reported type with 30 studies, whereas a considerably lower number of studies reported values for inter-day (three studies) and inter-season (one study) reliability. The ICC (29 studies) and CV (22 studies) were the most reported metrics, followed by SEM (four studies), Cronbach’s alpha (three studies), Pearson’s r (two studies) and MDC (one study). Countermovement jump (27 studies), SJ (15 studies) and SLCMJ (five studies) represented the tests with the highest availability of reliability values. Specifically, intra-day reliability for CMJ height ranged from 0.80 to 0.99 (ICC) and from 1.8 to 15% (CV), with a SEM that ranged from 0.6 to 1.4 cm. In contrast, the only study that investigated the inter-day reliability in CMJ height reported values of 0.83 (ICC) and 4.3% (CV), with a SEM of 1.7 cm. With respect to SJ height, intra-day reliability ranged from 0.75 to 0.99 (ICC) and from 2.12 to 13.2% (CV), with a SEM of 0.6 cm. Similar to CMJ, only one study examined inter-day reliability, reporting an ICC value of 0.89, a CV of 3.7% and a SEM of 1.4 cm. Last, only intra-day reliability was reported for SLCMJ height. In particular, ICC exhibited a range from 0.74 to 0.99, a CV from 1.98 to 9.63% and a SEM from 0.3 to 1 cm.

### Normative Values for Strength in Elite Male Soccer Players

#### Knee Extensor Isokinetic Strength via Isokinetic Dynamometry

Table 4 provides the normative values for the knee extensor isokinetic strength test. A range of different angular velocities was observed in the studies that reported normative values in the knee extensor isokinetic strength testing. However, the majority of the studies that reported normative values were conducted at 60°/s. In senior professionals, the mean values ranged from 212.9 to 364 Nm in peak concentric torque (32 studies), from 2.45 to 3.62 Nm/kg in relative peak concentric torque (15 studies) and from 54.0 to 65.5% for the conventional strength hamstrings-to-quadriceps ratio (17 studies). In elite youth, the mean values ranged from 208 to 331 in peak concentric torque (six studies). In terms of relative peak concentric torque, only one study reported normative values, with a value of 3.14 Nm/kg. Finally, the mean values of conventional strength hamstrings-to-quadriceps ratio ranged from 50 to 60.5 in elite youth soccer players (three studies).

**Table 4:** Normative values for peak concentric torque, relative peak concentric torque and conventional strength hamstrings/quadriceps ratio during the knee extensors isokinetic strength test

Study	Playing standard	Playing position/subgroup	Dynamometer type	Angular velocity (°/s)	Peak concentric torque (Nm)	Relative peak concentric torque (Nm/kg)	Conventional strength hamstrings/quadriceps ratio (%)
Maestroni et al. [66]	Senior professionals	Pre-injury and healthy group	Biodex system	60		3.13 ± 0.40	
Cossich et al. [85]	Senior professionals	All	Humac Norm	60	250 ± 43		
Misjuk and Rannama [94]	Senior professionals	All	Humac Norm	60	231.3 ± 31.0	3.01 ± 0.32	
			Humac Norm	300	121.1 ± 16.6	1.58 ± 0.18	
Scoz et al. [123]	Senior professionals	All (excluding goalkeepers)	Cybex Humac Norm	60	302.0 ± 47.9		
Beato et al. [18]	Senior professionals	All	Cybex Norm	60	282.9 ± 48.6		60 ± 8.6
			Cybex Norm	300	144.3 ± 22.3		66.5 ± 11
	Elite youth	All	Cybex Norm	60	242.5 ± 38.9		55.5 ± 9.5
			Cybex Norm	300	125.5 ± 18.3		63.5 ± 9.5
Śliwowski et al. [132]	Senior professionals	All	Biodex system	60		3.21 ± 0.40	59.2 ± 8.0
Eustace et al. [133]	Senior professionals	All	Biodex system	60	244.5 ± 31.6		
			Biodex system	180	201.4 ± 24.5		
			Biodex system	270	174.3 ± 25.5		
	Elite youth	All	Biodex system	60	210.9 ± 34.8		
			Biodex system	180	164.7 ± 24		
			Biodex system	270	137.2 ± 26.9		
Shalaj et al. [101]	Senior professionals	All	Biodex system	60	224.6 ± 34.4		58.4 ± 9.9
			Biodex system	240	142.8 ± 22.4		71.2 ± 13.1
Correia et al. [105]	Senior professionals	Uninjured group	Biodex system	60	223.4 ± 25.6		55.0 ± 12.0
			Biodex system	180	154.9 ± 12.7		60.0 ± 9.0
Ribeiro-Alvares et al. [106]	Senior professionals and elite youth	All	Biodex system	60	273.9 ± 40.8		55.0 ± 6.0
Michaelides et al. [137]	Senior professionals	All	Humac Norm	60	239.8 ± 42.6		
			Humac Norm	300	128.8 ± 28.6		
Van Dyk et al. [140]	Senior professionals	All (pre-injury)	Biodex system	60	239.1 ± 44.0		
			Biodex system	300	138.1 ± 24.4		
López-Valenciano et al. [142]	Senior professionals	All	Biodex system	60		2.45 ± 0.45	
			Biodex system	180		1.7 ± 0.3	
			Biodex system	240		1.5 ± 0.3	
			Biodex system	300		1.35 ± 0.3	
Almeida et al. [148]	Senior professionals	Control group	Biodex system	60	358 ± 44.2		
Coratella et al. [149]	Elite youth	All	Cybex Norm	30		3.3 ± 0.36	
			Cybex Norm	300		1.7 ± 0.17	
Śliwowski et al. [152]	Elite youth	All	Biodex system	60	331.3 ± 32.9		
Van Dyk et al. [153]	Senior professionals	All	Biodex system	60	235.1 ± 45.4		
			Biodex system	300	136.3 ± 26.8		
Buśko et al. [154]	Senior professionals	All (strikers)	Biodex system	60	243.7 ± 36		59 ± 10.5
			Biodex system	180	171.2 ± 29.2		64 ± 13.5
			Biodex system	300	129.2 ± 29.7		74 ± 20.5
Bakken et al. [155]	Senior professionals	All	Biodex system	60	235.1 ± 46.4	3.27 ± 0.56	54 ± 9
			Biodex system	300	134.9 ± 25.7	1.87 ± 0.30	72 ± 11.5
Van Dyk et al. [42]	Senior professionals	All	Biodex system	60	236.2 ± 46.6	3.28 ± 0.6	
			Biodex system	300	136.1 ± 28.6	1.88 ± 0.4	
Śliwowski et al. [160]	Senior professionals	All (excluding goalkeepers)	Biodex system	60		3.27 ± 0.35	58.4 ± 7.13
Van Dyk et al. [170]	Senior professionals	All	Biodex system	60	231.1 ± 41.4		
			Biodex system	300	133.4 ± 25.6		
Carvalho et al. [173]	Senior professionals	First league group	Technogym	60	257 ± 47.5		61.5 ± 10.5
		Second league group	Technogym	60	234.5 ± 36		58.5 ± 9.5
Tsiokanos et al. [179]	Senior professionals	All (excluding goalkeepers)	Cybex system	30	309.7 ± 28.4	4.1 ± 0.38	
			Cybex system	60	276.2 ± 25.8	3.62 ± 0.36	
			Cybex system	180	182.7 ± 18.2	2.37 ± 0.25	
Bogdanis and Kalapotharakos [181]	Senior professionals	All	Biodex system	60	291 (250–335)	54.35 (46.2–61.3)	
			Biodex system	180	201 (176–231)		
			Biodex system	300	159.5 (143–173)		
Enright et al. [177]	Elite youth	All	Kin-Com	60	217 ± 28.5		
			Kin-Com	180	198.5 ± 21		
Ruas et al. [178]	Senior professionals	All	Cybex Norm	60	260.8 ± 40.6		60 ± 10
Ardern et al. [184]	Senior professionals	All	Humac Norm	60	220.7 ± 57.4		64.5 ± 15
			Humac Norm	240	116.7 ± 26.0		83.5 ± 20
Ruas et al. [186]	Senior professionals	All	Cybex Norm	60	259.8 ± 37.5		61 ± 11.7
Portella et al. [197]	Senior professionals	All	Biodex system	60	302 ± 47.9		
Rebelo et al. [200]	Elite youth	Elite U19 group (excluding goalkeepers)	Biodex system	90	212.5 ± 32.9		
Menzel et al. [201]	Senior professionals	All	Biodex system	60		3.39 ± 0.54	
			Biodex system	180		2.34 ± 0.30	
			Biodex system	300		1.72 ± 0.24	
Silva et al. [202]	Senior professionals	All	Biodex system	90	231.5 ± 30.5		
Daneshjoo et al. [209]	Elite youth	All	Biodex system	60			50 ± 6.83
			Biodex system	180			53.3 ± 13.16
			Biodex system	300			74.3 ± 25
Whiteley et al. [210]	Senior professionals	All	Biodex system	60	230.9 ± 41.9	3.24 ± 0.49	
			Biodex system	300	131.3 ± 25.6	1.86 ± 0.29	
Greco et al. [211]	Senior professionals	All	Biodex system	60	255.3 ± 29.4		
Silva et al. [213]	Senior professionals	All	Biodex system	90	243.9 ± 53.0		54.9 ± 6.1
Cotte and Chatard [220]	Senior professionals	All	Cybex Norm	60	243 ± 41.5	2.86 ± 0.33	
			Cybex Norm	180	175 ± 25	2.08 ± 0.20	
			Cybex Norm	240	154.5 ± 22	1.83 ± 0.23	
			Cybex Norm	300	132 ± 19.5	1.56 ± 0.18	
Henderson et al. [221]	Senior professionals	All	Biodex system	60	262.5 ± 41.5	3.50 ± 0.67	60 ± 9
			Biodex system	180	197 ± 29	2.64 ± 0.53	63 ± 11
			Biodex system	300	158 ± 23.5	2.12 ± 0.42	64 ± 10
Lehance et al. [21]	Senior professionals	Professional group	Cybex Norm	60	224.2 ± 38.8	2.96 ± 0.40	60.5 ± 7.0
			Cybex Norm	240	136.9 ± 18.7	1.78 ± 0.21	70.5 ± 15.5
	Elite youth	U21 group	Cybex Norm	60	231.7 ± 30.4	3.14 ± 0.47	60.5 ± 7.5
			Cybex Norm	240	133.3 ± 17.6	1.86 ± 0.23	74 ± 14.5
Hoshikawa et al. [229]	Senior professionals	All	Biodex system	60	212.9 ± 32.9		56.5 ± 7.0
			Biodex system	180	158.7 ± 20.9		64.5 ± 8.0
Voutselas et al. [235]	Senior professionals	All	Cybex Norm	60	213.3 ± 38.2		65.5 ± 9
Kalapotharakos et al. [236]	Senior professionals	All	Cybex system	60	245 ± 37.2		
Kraemer et al. [238]	Elite youth	All	Cybex system	60	208 ± 7.9		
Ozcakar [241]	Senior professionals	All	Biodex system	60	364 ± 44.5		
			Biodex system	240	197.8 ± 29.1		
Gür et al. [246]	Senior professionals	All	Cybex system	30	233.5 ± 35		
			Cybex system	180	157 ± 21.5		
			Cybex system	240	144 ± 22		
			Cybex system	300	132 ± 18		
	Elite youth	All	Cybex system	30	226 ± 23		
			Cybex system	180	145 ± 12.5		
			Cybex system	240	132.5 ± 13		
			Cybex system	300	121.5 ± 13.5		
Aagaard et al. [248]	Senior professionals	All	Kin-Com	30	253 ± 51		
			Kin-Com	120	202.2 ± 36.7		
			Kin-Com	240	145.2 ± 27.7		
Chin et al. [249]	Senior professionals	All	Cybex system	60		2.72 ± 0.36	60
Poulmedis [251]	Senior professionals	All	Cybex system	30	247 ± 29		
			Cybex system	90	191 ± 35		
			Cybex system	180	126 ± 26		
Rhodes et al. [252]	Elite youth	All	Cybex system	30	246.3 ± 36.6		

Data are presented as mean ± standard deviation

*U19* under 19 years of age, *U21* under 21 years of age

#### Knee Flexor Isokinetic Strength via Isokinetic Dynamometry

Normative values reported for the knee flexor isokinetic strength test can be found in Table 5. As with knee extensor isokinetic strength testing, a variety of different velocities were used in the studies that reported normative values for knee flexor isokinetic strength test. Angular velocity of 60°/s had the greatest number of available normative data. For senior professionals at 60°/s, the mean values for peak concentric torque ranged from 113.2 to 190.5 Nm (30 studies), from 153 to 213.4 Nm for peak eccentric torque (15 studies) and from 1.2 to 2.1 Nm/kg for relative peak concentric torque (11 studies). Conversely, elite youth players had average peak concentric torque values that ranged from 114 to 187.4 Nm (four studies). Only two studies reported a normative value in elite youth for eccentric peak torque (range 149–177.1 Nm). No study reported relative peak concentric torque values for elite youth soccer players.

**Table 5:** Normative values for peak concentric torque, peak eccentric torque and relative peak concentric torque during the knee flexors isokinetic strength test

Study	Playing standard	Playing position/subgroup	Dynamometer type	Angular velocity (°/s)	Peak concentric torque (Nm)	Peak eccentric torque (Nm)	Relative peak concentric torque (Nm/kg)
Maestroni et al. [66]	Senior professionals	Pre-injury and healthy group	Biodex system	60			1.74 ± 0.26
Misjuk and Rannama [94]	Senior professionals	All	Humac Norm	60	145.7 ± 22.3		1.90 ± 0.23
			Humac Norm	300	83 ± 16.7		1.08 ± 0.17
Scoz et al. [123]	Senior professionals	All (excluding goalkeepers)	Cybex Humac Norm	60	184.4 ± 30.6	213 ± 40.1	
Beato et al. [18]	Senior professionals	All	Cybex Norm	60	171.3 ± 38.8	213.4 ± 62.1	
			Cybex Norm	300	97 ± 17.7		
	Elite youth	All	Cybex Norm	60	134.4 ± 25.8	177.1 ± 37.2	
			Cybex Norm	300	80.2 ± 13.8		
Śliwowski et al. [132]	Senior professionals	All	Biodex system	60			1.90 ± 0.29
Eustace et al. [133]	Senior professionals	All	Biodex system	60		192.4 ± 31.5	
			Biodex system	180		186.9 ± 27.1	
			Biodex system	270		186.8 ± 18.8	
	Elite youth	All	Biodex system	60		149 ± 27.5	
			Biodex system	180		157.2 ± 28.8	
			Biodex system	270		156.9 ± 29.5	
Shalaj et al. [101]	Senior professionals	All	Biodex system	30		120.3 ± 44.3	
			Biodex system	60	129.7 ± 21.2		
			Biodex system	120		97.6 ± 46.5	
			Biodex system	240	100.7 ± 21.4		
Correia et al. [105]	Senior professionals	Uninjured group	Biodex system	60	123.1 ± 26.5	161.8 ± 50.8	
			Biodex system	180	92.4 ± 15.8	175.3 ± 54.9	
Ribeiro-Alvares et al. [106]	Senior professionals and elite youth	All	Biodex system	60	150.0 ± 22.5	207.9 ± 39.6	
Michaelides et al. [137]	Senior professionals	All	Humac Norm	60	173.6 ± 31.8		
			Humac Norm	300	103.6 ± 23.6		
Van Dyk et al. [140]	Senior professionals	All (pre-injury)	Biodex system	60	119.1 ± 24.6	176.9 ± 46.8	
			Biodex system	300	98.6 ± 19.0		
López-Valenciano et al. [142]	Senior professionals	All	Biodex system	60			1.2 ± 0.2
			Biodex system	180			0.9 ± 0.2
			Biodex system	240			0.9 ± 0.2
			Biodex system	300			0.9 ± 0.2
Almeida et al. [148]	Senior professionals	Control group	Biodex system	60	190.5 ± 18.5		
Coratella et al. [149]	Elite youth	All	Cybex Norm	30			1.85 ± 0.24
			Cybex Norm	300			1.06 ± 0.22
Śliwowski et al. [152]	Elite youth	All	Biodex system	60	187.4 ± 23.6		
Van Dyk et al. [153]	Senior professionals	All	Biodex system	60	128.6 ± 26.1	185.5 ± 39.5	
			Biodex system	300	98.6 ± 21.4		
Buśko et al. [154]	Senior professionals	All (strikers)	Biodex system	60	143.3 ± 31.3		
			Biodex system	180	108.2 ± 24.1		
			Biodex system	300	93.2 ± 26.2		
Bakken et al. [155]	Senior professionals	All	Biodex system	60	126.7 ± 27.2	203.8 ± 42.6	1.76 ± 0.33
			Biodex system	300	96.4 ± 19.6		1.33 ± 0.24
Lee et al. [156]	Senior professionals	All	Biodex system	30		151.3 ± 26.9	
			Biodex system	60	113.2 ± 19.7		
Van Dyk et al. [42]	Senior professionals	All	Biodex system	60	125.9 ± 25.8	204.2 ± 41.5	1.75 ± 0.3
			Biodex system	300	96.3 ± 18.6		1.34 ± 0.2
Śliwowski et al. [160]	Senior professionals	All (excluding goalkeepers)	Biodex system	60			1.91 ± 0.28
Van Dyk et al. [170]	Senior professionals	All	Biodex system	60	124.4 ± 31.1	182.3 ± 39.8	
			Biodex system	300	94.8 ± 21.2		
Carvalho et al. [173]	Senior professionals	First league group	Technogym	60	155.5 ± 28	178.5 ± 60	
		Second league group	Technogym	60	136.5 ± 25.5	163.5 ± 32.5	
Bogdanis and Kalapotharakos [181]	Senior professionals	All	Biodex system	60	158 (143–173)		
			Biodex system	180	125.5 (117–135)		
			Biodex system	300	110 (101–121)		
Enright et al. [177]	Elite youth	All	Kin-Com	60	114 ± 20.5		
			Kin-Com	120		144.5 ± 22	
			Kin-Com	180	110.5 ± 17		
Ruas et al. [178]	Senior professionals	All	Cybex Norm	60	155.9 ± 32.3	202.1 ± 41.6	
Ardern et al. [184]	Senior professionals	All	Humac Norm	30		158.7 ± 50.3	
			Humac Norm	60	140 ± 31.7		
			Humac Norm	120		156.7 ± 42.2	
			Humac Norm	240	98.7 ± 22		
Ruas et al. [186]	Senior professionals	All	Cybex Norm	60	157.2 ± 31.9	201.1 ± 40.1	
Booysen et al. [188]	Senior professionals	Professional group	Biodex system	90		256.5 (230–293)	
Portella et al. [197]	Senior professionals	All	Biodex system	60	178.1 ± 35.2		
Rebelo et al. [200]	Elite youth	Elite U19 group (excluding goalkeepers)	Biodex system	90	112.5 ± 19.4		
Silva et al. [202]	Senior professionals	All	Biodex system	90	126.5 ± 16.5		
Whiteley et al. [210]	Senior professionals	All	Biodex system	60	124.3 ± 24.1	181.5 ± 38.7	1.76 ± 0.29
			Biodex system	300	96.4 ± 22.5		1.35 ± 0.25
Greco et al. [211]	Senior professionals	All	Biodex system	60	153.6 ± 20.6		
Silva et al. [213]	Senior professionals	All	Biodex system	90	135.9 ± 23.9		
Cotte and Chatard [220]	Senior professionals	All	Cybex Norm	60	152 ± 30.5		1.79 ± 0.29
			Cybex Norm	180	111 ± 19.5		1.30 ± 0.18
			Cybex Norm	240	101 ± 18		1.19 ± 0.17
			Cybex Norm	300	86.5 ± 15.5		1.02 ± 0.15
Henderson et al. [221]	Senior professionals	All	Biodex system	60	156.5 ± 31		2.10 ± 0.48
			Biodex system	180	123 ± 23		1.65 ± 0.35
			Biodex system	300	101.5 ± 21		1.36 ± 0.33
Lehance et al. [21]	Senior professionals	Professional group	Cybex Norm	30		200.1 ± 52.4	
			Cybex Norm	60	136.8 ± 34.1		
			Cybex Norm	120		197.6 ± 44.2	
			Cybex Norm	240	100.8 ± 12.3		
	Elite youth	U21 group	Cybex Norm	30		194.2 ± 44.5	
			Cybex Norm	60			
			Cybex Norm	120		196.8 ± 39.8	
			Cybex Norm	240			
Hoshikawa et al. [229]	Senior professionals	All	Biodex system	60	120.2 ± 22.4		
			Biodex system	180	102.1 ± 18.9		
Voutselas et al. [235]	Senior professionals	All	Cybex Norm	60	144.6 ± 26.6		
Kraemer et al. [238]	Elite youth	All	Cybex system	60	135.7 ± 5.81		
Askling et al. [240]	Senior professionals	All	Kin-Com	60	130.5 ± 23	153 ± 24.5	
Ozcakar [241]	Senior professionals	All	Biodex system	60	183.5 ± 29.2		
			Biodex system	240	130.9 ± 19.1		
Gür et al. [246]	Senior professionals	All	Cybex system	30	130.5 ± 18.5	143.5 ± 35.5	
			Cybex system	180	97 ± 15.5	154.5 ± 28	
			Cybex system	240	87.5 ± 15.5	156 ± 29	
			Cybex system	300	82 ± 17.5	158 ± 24	
	Elite youth	All	Cybex system	30	124.5 ± 14	138 ± 27.5	
			Cybex system	180	87 ± 15	138 ± 20.5	
			Cybex system	240	76 ± 8.5	137 ± 20.5	
			Cybex system	300	71.5 ± 9	141 ± 22.5	
Aagaard et al. [248]	Senior professionals	All	Kin-Com	30	120.5 ± 25.7	143.2 ± 34.7	
			Kin-Com	120	102 ± 19.5	155.2 ± 39.4	
			Kin-Com	240	73.7 ± 15.2	150.2 ± 30.6	
Chin et al. [249]	Senior professionals	All	Cybex system	60			1.65 ± 0.2
Poulmedis [251]	Senior professionals	All	Cybex system	30	146 ± 12		
			Cybex system	90	125 ± 26		
			Cybex system	180	93 ± 23		
Rhodes et al. [252]	Elite youth	All	Cybex system	30	120.8 ± 16.1		

Data are presented as mean ± standard deviation

*U19* under 19 years of age, *U20* under 20 years of age

#### Knee Flexor Eccentric Strength via Nordic Hamstring Exercise

Results of the peak force (N) attained by elite soccer players can be found in Table 6. As can be observed, different equipment has been used to assess eccentric knee flexor strength. More specifically, the range of the average values in senior professionals was from 277.5 to 403.7 N (seven studies). Only two studies reported values for peak force in elite youth soccer players. The different devices employed yielded extremely different values, with one reporting a value of 338.2 N and the other 636.5 N.

**Table 6:** Normative values for peak force during the Nordic hamstring strength test

Study	Playing standard	Playing position/subgroup	Equipment	Peak force (N)
Cadu et al. [95]	Senior professionals	All	Nordbord	357.3 ± 95.0
Bishop et al. [253]	Senior professionals	All	Nordbord	399.1 ± 74.0
Suarez-Arrones et al. [107]	Elite youth	All	Acceleration leg curl/extension, Neuroexcellence	636.5 ± 110.5
Capaverde et al. [121]	Senior professionals and elite youth	All	Nordic assessment device	364.1 ± 66.4
Ribeiro-Alvares et al. [122]	Senior professionals	All	Nordic assessment device	378.2 ± 61.77
Grazioli et al. [131]	Senior professionals	All	Nordic assessment device	403.7 ± 53.6
Moreno-Pérez et al. [104]	Elite youth	All	Nordic assessment device	338.2 ± 45.2
Van Dyk et al. [153]	Senior professionals	All	Nordic assessment device	298.6 ± 72.3
Van Dyk et al. [42]	Senior professionals	All	Nordic assessment device	304.4 ± 66.3
Timmins et al. [45]	Senior professionals	All	Nordic assessment device	277.5 ± 73.1

Data are presented as mean ± standard deviation

### Normative Values for Power in Elite Male Soccer Players

Normative values for the CMJ, SJ, and VJ test are presented in Tables 7, 8 and 9, respectively. For CMJ, the average values of jump height observed in senior professional soccer players ranged from 33.6 to 57.2 cm across 54 studies, while the mean values ranged from 34.8 to 58.6 cm across 33 studies in elite youth soccer players. In terms of relative peak power during CMJ, the average values in senior professional soccer players ranged from 26.3 to 54.5 W/kg across four studies. However, only one study reported the relative peak power value in elite youth, which was 55.1 W/kg. In addition, the average peak power values in senior professionals ranged from 3474 to 5029 W (range 3474–5029) [four studies], while the only study that reported a value in elite youth yielded a value of 3778 W. For SJ, the average jump height in senior players ranged from 29.8 to 44.1 cm (23 studies), whereas it ranged from 34.3 to 52.8 cm in youth (16 studies). Last, the average VJ jump height values in senior players ranged from 41.1 to 56.4 cm across 13 studies, while the mean values in elite youth ranged from 41.6 to 65 cm across 13 studies.

**Table 7:** Normative values for jump height, peak power and relative peak power during the countermovement jump

Study	Playing standard	Playing position/subgroup	Equipment used (method)	Jump height (cm)	Relative peak power (W/kg)	Peak power (W)
Maestroni et al. [66]	Senior professionals	Pre-injury and healthy group	Force plates (impulse-momentum)	36.9 ± 5.5	52.4 ± 5.6	
Espada et al. [84]	Senior professionals	All	Photoelectric system	38.4 ± 5.9		
Byrkjedal et al. [86]	Senior professionals	All	Force plates (impulse-momentum)	40.6 ± 5.3	32.2 ± 3.8	
Bishop et al. [64]	Elite youth	All	Force plates (impulse-momentum)	40.1 ± 3.5		
Guerra et al. [88]	Senior professionals	All	Contact platform	42.2 ± 3.9		
Bongiovanni et al. [91]	Senior professionals	High performers	Force plates	52.1 ± 5.1		5029.8 ± 407.9
		Low performers		47.9 ± 4.6		4189 ± 396.9
Boraczyński et al. [93]	Senior professionals	All	Tensometric platform	46 ± 4.7		
Misjuk and Rannama [94]	Senior professionals	All	Force plates (flight time)	35 ± 5.2		
Schons et al. [96]	Senior professionals	All (senior professionals)	Contact mat (flight time)	39.7 ± 4.6		
Freitas et al. [97]	Elite youth	All	Contact platform	42.9 ± 4.6		
Arregui-Martin et al. [98]	Elite youth	All	Photoelectric system (Optojump)	43.7 ± 4.3		
Querido and Clemente [100]	Elite youth	All	Contact platform (flight time)	42.9 ± 4.7		
Shalaj et al. [101]	Senior professionals	All	Force plates	44.6 ± 4.9		
Ribeiro et al. [109]	Elite youth	All	Contact platform (flight time)	38.5 ± 2.8		
Krespi et al. [103]	Elite youth	All	Force plates	52.3 ± 5.2		
Stern et al. [14]	Elite youth	All	Contact mat	58.6 ± 6.7		
Dolci et al. [113]	Elite youth	All	Force plates		48.3 ± 6.8	
Bishop et al. [63]	Elite youth	U23 group	Photoelectric system (Optojump)	38.8 ± 4.0		
		U18 group		37.7 ± 4.3		
Rodrigues Júnior et al. [120]	Senior professionals	All	Force plates (impulse-momentum)	41.1 ± 3.5		
Loturco et al. [130]	Elite youth	All	Contact platform	44.4 ± 5.5		
Grazioli et al. [131]	Senior professionals	All	Contact platform	43.4 ± 4.9		
Papadakis et al. [65]	Senior professionals	All	Photoelectric system (Optojump)	41.2 ± 5.0		
Arcos et al. [134]	Senior professionals	Senior group	Photoelectric system (Optojump)	42.4 ± 5.5		
	Elite youth	U19 group		45.4 ± 4.3		
		U17 group		45.9 ± 5.3		
Saidi et al. [138]	Senior professionals	All		39.2 ± 2.4		
Northeast et al. [59]	Senior professionals	All	Force plates	39.0 ± 4.0	54.5 ± 5.3	4229.1 ± 602.9
Rago et al. [143]	Senior professionals	All	Accelerometric system	36.4 ± 3.2		
Loturco et al. [144]	Elite youth	All	Contact mat	44.7 ± 5.1		
Los Arcos and Martins [147]	Elite youth	All	Contact mat	44.7 ± 4.0		
Enright et al. [80]	Elite youth	All	Jump mat	40.8 ± 4.2		
Coratella et al. [149]	Elite youth	All	Photoelectric system (Optojump)	40.1 ± 4.7		
Gil et al. [151]	Senior professionals	All	Contact platform	40.4 ± 3.7		
Śliwowski et al. [152]	Elite youth	All	Photoelectric system (Optojump)	36.7 ± 4.3		
Buśko et al. [154]	Senior professionals	All (strikers)	Force plates	39.9 ± 4.7	26.3 ± 5.3	3474.7 ± 1111.5
Haugen [157]	Senior professionals	All	Force plates	38.6 ± 3.9		
Otero-Esquina et al. [13]	Elite youth	All	Force plates (impulse-momentum)	35.2 ± 3.7	53.5 ± 4.9	
Loturco et al. [164]	Elite youth	All	Contact platform	38.6 ± 4.7		
Pareja-Blanco et al. [166]	Senior professionals	All	Photoelectric system (Optojump)	34.6 ± 4.4		
Krommes et al. [167]	Senior professionals	All	Force plates (impulse-momentum)	44.1 ± 4.6		
Yanci and Los Arcos [168]	Elite youth	All	Jumping mat	43.4 ± 4.0		
De Hoyo et al. [12]	Elite youth	All	Photoelectric system (Optojump)	36.1 ± 3.7		
Fessi et al. [171]	Senior professionals	All	Force plates	41.4 ± 3.4		
Rey et al. [174]	Senior professionals	All	Force plates	40.3 ± 3.7		
Martinez-Santos et al. [62]	Elite youth	All	Jumping mat	45.4 ± 4.7		
Loturco et al. [175]	Senior professionals	All	Contact mat	42.0 ± 2.8		
Enright et al. [177]	Elite youth	All	Jump mat	39.9 ± 3.3		
Loturco et al. [75]	Senior professionals	All	Contact mat	41.5 ± 3.7		
Pareja-Blanco et al. [180]	Senior professionals	All	Photoelectric system (Optojump)	33.6 ± 3.6		
Gil et al. [182]	Senior professionals	All	Contact platform	41.3 ± 4.1		
Loturco et al. [185]	Elite youth	All	Force plates	42.7 ± 3.9		
Arcos et al. [187]	Elite youth	All	Contact mat	42.9 ± 4.2		
Booysen et al. [188]	Senior professionals	Professional group	Contact mat	36.1 (34.0–38.1)		
Edholm et al. [191]	Senior professionals	All	Photoelectric system	39.0 ± 2.9		
Brocherie et al. [192]	Senior professionals	All	Force plates (impulse-momentum)	41.7 ± 4.1		
Arcos et al. [194]	Senior professionals	All	Contact mat	44.0 ± 4.9		
Koundourakis et al. [195]	Senior professionals	All	Jumping mat	42.6 ± 4.2		
Koundourakis et al. [196]	Senior professionals	All	Jumping mat	39.6 ± 3.0		
Haugen et al. [199]	Senior professionals	National team group	Force plates	39.4 ± 5.2		
		First division group		39.0 ± 4.6		
		Second division group		38.8 ± 4.6		
	Elite youth	Elite youth national team group		39.0 ± 4.6		
Rebelo et al. [200]	Elite youth	Elite U19 group (excluding goalkeepers)	Jumping mat	38.9 ± 4.6		
Menzel et al. [201]	Senior professionals	All	Force plates			
Silva et al. [202]	Senior professionals	All	Jumping platform	42.4 ± 4.4		
Castagna and Castellini [204]	Elite youth	U21 group	Photoelectric system (Optojump)	40.3 ± 4.3		
		U20 group		40.2 ± 4.7		
Lago-Ballesteros [205]	Senior professionals	All	Contact platform	39.3 ± 3.5		
Boone et al. [207]	Senior professionals	All (excluding goalkeepers)	Jumping mat	43.2 ± 3.7		
Silva et al. [213]	Senior professionals	All	Jumping platform	42.2 ± 4.4		
Helgerud et al. [215]	Senior professionals	All	Force plates	57.2 ± 4.8		
Rønnestad et al. [216]	Senior professionals	All	Force plates	39.3 ± 1.6		
Faude et al. [218]	Senior professionals	All	Jumping platform	36.7 ± 4.0		
	Elite youth	All				
López-Segovia et al. [223]	Elite youth	All	Contact platform	34.8 ± 5.1		
Henderson et al. [221]	Senior professionals	All	Force plates	40.0 ± 5.0		
Till and Cooke [224]	Elite youth	All	Jump mat	40.5 ± 4.8		
Mujika et al. [225]	Elite youth	All	Contact platform	45.6 ± 3.7		
Mujika et al. [226]	Senior professionals	All	Contact platform	43.7 ± 2.2		
	Elite youth	All		43.9 ± 4.8		
Sporis et al. [227]	Senior professionals	All	Force plates	45.1 ± 1.7		
Ronnestad et al. [231]	Senior professionals	All	Force plates	35.2 ± 1.2		
Kalapotharakos et al. [236]	Senior professionals	All	Contact platform	43.8 ± 4.3		
Chamari et al. [239]	Elite youth	All	Force plates	51.3 ± 6.7	55.1 ± 5.7	3878 ± 553
Ostojic [237]	Senior professionals	Professional group	Contact platform	49.9 ± 7.5		
Arnason et al. [19]	Senior professionals	All	Contact mat	39.2 ± 5.0		
Ozcakar [241]	Senior professionals	All	Jumping mat	44.8 ± 4.5		
Casajús [243]	Senior professionals	All	Contact platform	40.8 ± 2.7		
Cometti et al. [244]	Senior professionals	All (Division 1 and 2 group)	Jumping mat	40.6 ± 4.7		

Data are presented as mean ± standard deviation

*U17* under 17 years if age, *U18* under 18 years of age, *U19* under 19 years of age, *U20* under 20 years of age, *U21* under 21 years of age, *U23* under 23 years of age

**Table 8:** Normative values for jump height during the squat jump

Study	Playing standard	Playing position/subgroup	Equipment used (method)	Jump height (cm)
Espada et al. [84]	Senior professionals	All	Photoelectric system	36.4 ± 7.0
Guerra et al. [88]	Senior professionals	All	Contact platform	37.6 ± 4.6
Keiner et al. [15]	Elite youth	All	Contact mat	38.3 ± 2.9
Schons et al. [96]	Senior professionals	All (senior professionals)	Contact mat (flight time)	37.9 ± 5.0
Freitas et al. [97]	Elite youth	All	Contact platform	39.5 ± 3.6
Querido and Clemente [100]	Elite youth	All	Contact platform (flight time)	42.1 ± 4.1
Ribeiro et al. [109]	Elite youth	All	Contact platform (flight time)	34.3 ± 3.2
Cardoso De Araújo et al. [127]	Senior professionals	All (senior professionals)	Force plates (impulse-momentum)	35.3 ± 3.9
Grazioli et al. [131]	Senior professionals	All	Contact platform	38.7 ± 4.0
Papadakis et al. [65]	Senior professionals	All	Photoelectric system (Optojump)	39.2 ± 5.0
Hoppe et al. [17]	Elite youth	U21 group	Force plates (impulse-momentum)	38.2 ± 2.3
		U19 group		35.5 ± 1.2
Krespi et al. [103]	Elite youth	All	Force plates	41.6 ± 4.6
Saidi et al. [138]	Senior professionals	All		35.5 ± 3.9
Loturco et al. [144]	Elite youth	All	Contact mat	43.7 ± 4.7
Coratella et al. [149]	Elite youth	All	Photoelectric system (Optojump)	34.5 ± 4.2
Enright et al. [80]	Elite youth	All	Jump mat	38.7 ± 4.3
Gil et al. [151]	Senior professionals	All	Contact platform	40.5 ± 3.7
Śliwowski et al. [152]	Elite youth	All	Photoelectric system (Optojump)	35.2 ± 4.0
Loturco et al. [164]	Elite youth	All	Contact platform	37.4 ± 4.6
Loturco et al. [175]	Senior professionals	All	Contact mat	38.0 ± 3.7
Enright et al. [177]	Elite youth	All	Jump mat	38.4 ± 4.3
Loturco et al. [75]	Senior professionals	All	Contact mat	39.6 ± 3.3
Koundourakis et al. [195]	Senior professionals	All	Jumping mat	41.1 ± 3.6
Koundourakis et al. [196]	Senior professionals	All	Jumping mat	37.7 ± 3.1
Rebelo et al. [200]	Elite youth	Elite U19 group (excluding goalkeepers)	Jumping mat	37.7 ± 4.8
Castagna and Castellini [204]	Elite youth	U21 group	Photoelectric system (Optojump)	37.0 ± 3.9
		U20 group		38.0 ± 4.9
Lago-Ballesteros [205]	Senior professionals	All	Contact platform	37.7 ± 2.6
Boone et al. [207]	Senior professionals	All (excluding goalkeepers)	Jumping mat	40.4 ± 3.5
Rønnestad et al. [216]	Senior professionals	All	Force plates	37.1 ± 1.1
Henderson et al. [221]	Senior professionals	All	Force plates	40.0 ± 5.0
Sporis et al. [227]	Senior professionals	All	Force plates	44.1 ± 1.3
Ronnestad et al. [231]	Senior professionals	All	Force plates	29.8 ± 1.0
Chamari et al. [232]	Elite youth	All	Force plates	52.8 ± 5.5
Rampinini et al. [234]	Senior professionals	All	Photoelectric system (Optojump)	36.6 ± 3.8
Arnason et al. [19]	Senior professionals	All	Contact mat	39.2 ± 5.0
Ozcakar [241]	Senior professionals	All	Jumping mat	41.7 ± 4.3
Casajús [243]	Senior professionals	All	Contact platform	39.2 ± 3.1
Cometti et al. [244]	Senior professionals	All (Division 1 and 2 group)	Jumping mat	36.2 ± 6.0

Data are presented as mean ± standard deviation

*U19* under 19 years of age, *U20* under 20 years of age, *U21* under 21 years of age

**Table 9:** Normative values for jump height during the vertical jump with free arms

Study	Playing standard	Playing position/subgroup	Equipment used (method)	Jump height (cm)
Lockie et al. [110]	Elite youth	All	Jumping mat	65 ± 8.0
Enes et al. [118]	Senior professionals	All	Contact platform	48.3 ± 6.1
Cardoso De Araújo et al. [127]	Senior professionals	All (senior professionals)	Force plates (impulse-momentum)	41.1 ± 4.5
Hoppe et al. [17]	Elite youth	U21 group	Force plates (impulse-momentum)	45.2 ± 2.5
		U19 group		41.6 ± 1.2
Arcos et al. [134]	Senior professionals	Senior group	Photoelectric system (Optojump)	50.0 ± 6.0
	Elite youth	U19 group		51.9 ± 4.9
		U17 group		51.3 ± 5.6
Buśko et al. [154]	Senior professionals	All (strikers)	Force plates	46.9 ± 4.6
Requena et al. [162]	Senior professionals	All	Contact platform	51.5 ± 5.9
Yanci and Los Arcos [168]	Elite youth	All	Jumping mat	49.1 ± 4.8
Fessi et al. [171]	Senior professionals	All	Force plates	48.1 ± 4.5
Rey et al. [174]	Senior professionals	All	Force plates	46.5 ± 6.4
Noon et al. [176]	Elite youth	All	Contact mat	43.0 ± 6.0
Arcos et al. [187]	Elite youth	All	Contact mat	50.6 ± 3.7
Brocherie et al. [192]	Senior professionals	All	Force plates (impulse-momentum)	48.0 ± 5.9
Lago-Ballesteros [205]	Senior professionals	All	Contact platform	46.8 ± 3.0
Daneshjoo et al. [206]	Elite youth	All	Chalk print	47.9 ± 6.2
Mujika et al. [225]	Elite youth	All	Contact platform	50.7 ± 5.2
Mujika et al. [226]	Senior professionals	All	Contact platform	50.1 ± 4.2
	Elite youth	All	Contact platform	51.8 ± 4.8
Chamari et al. [232]	Elite youth	All	Force plates	62.4 ± 5.6
Wisløff et al. [9]	Senior professionals	All	Force plates	56.4 ± 4.0
Helgerud et al. [242]	Elite youth	All	Force plates	53.4 ± 4.2
Casajús [243]	Senior professionals	All	Contact platform	46.7 ± 2.8
Wisløff et al. [247]	Senior professionals	All	Force plates	54.9 ± 5.3

*U17* under 17 years if age, *U19* under 19 years of age, *U21* under 21 years of age

## Discussion

The aims of this systematic review were to: (1) identify the tests and outcome variables used to evaluate strength and power in elite male soccer players; (2) provide normative values on the most common strength and power tests; and (3) report the reliability values of strength and power tests used in elite soccer. In summary, the large volume of studies included in this review (194 studies) is indicative of the high level of interest in strength and power assessment in soccer within the scientific community. A wide variety of tests were employed to assess strength and power, which was to be expected given the various time and financial constraints, as well as the different approaches to training and testing in soccer. A considerable amount of variability was also evident in the methods used to calculate the outcome variables of a test, as well as in the terminology used to describe the test. For instance, two distinct methods were identified in the calculation of the jump height (take-off velocity and flight-time method), while the terms “back squat” and “half-back squat” were used sometimes interchangeably. A total of 29 different tests were identified for strength assessment, of which the isokinetic strength test for knee extensors, the isokinetic strength test for knee flexors and the eccentric strength test for knee flexors were the most commonly used. However, 31 different tests were utilised to assess power, with CMJ, SJ and VJ being the most frequently employed. However, it is noteworthy that the majority of the studies included in this review failed to report reliability values, concealing valuable information that could assist in determining test accuracy and consistency.

### Testing Methods and Outcome Variables

As strength and power can support both performance enhancement [2, 9, 18] and injury risk minimisation [19–21], a valid and reliable assessment of strength and power ability can form the basis for effective prescription of training interventions. A plethora of strength and power tests were identified in our systematic review, reflecting a high level of interest in researching these attributes. However, this large disparity highlights the inherent complexities in the assessment of strength and power, as well as the lack of consensus on the optimal testing protocols for strength and power profiling in elite soccer players. This variation can be attributed to several factors, such as equipment availability and facilities, time constraints, safety and a competitive schedule among others [24]. Finally, cultural and philosophical differences may also have contributed to the wide range of different tests observed, as the included articles originated from 39 different countries.

#### Strength Assessment

Based on the results of this systematic review, isokinetic strength assessment of the knee extensor and flexor muscles represent the most popular testing methods to assess strength (58 and 55 studies, respectively). The large number of studies that have evaluated the strength of the knee extensors and flexors highlights the importance of these muscle groups in the execution of fundamental soccer-specific actions as well as in the prevention of common soccer injuries. In particular, knee extensors are involved in many soccer actions such as acceleration, deceleration, jumping and kicking, while knee flexors are highly recruited during running at higher velocities and provide additional support to the stabilisation of the knee joint during landing, deceleration and cutting actions [39]. In addition, the anterior cruciate ligament and the hamstring muscle group represent two of the most affected areas in soccer injuries [40, 41], which further highlights the necessity to assess knee extensor muscle function in soccer. The combination of the two measurements can be used for the calculation of the conventional strength ratio, enabling the determination of strength imbalances between knee extensor and knee flexor muscles. The conventional strength ratio is typically calculated by dividing the concentric peak torque of knee flexors by that of knee extensors [42, 43]. Its use is further supported by the findings of this systematic review, as it represents the second most frequent outcome variable in the isokinetic assessment of knee strength. It has been suggested that greater strength imbalances are associated with an increased risk of injury in the anterior cruciate ligament and hamstring muscle groups, although the overall research findings are inconsistent [20, 42, 44]. The assessment of peak concentric torque during knee flexor isokinetic strength and, subsequently, the conventional strength ratio, may fail to consider the main mechanism of hamstring strain injuries, where an eccentric muscle action occurs. This is further linked with increased demands of high-speed running (> 19.8 km/h) in modern soccer, where the hamstring muscles are subjected to additional eccentric loading. This appears to be the reason why the assessment of peak eccentric torque is another variable of interest (i.e. the second most investigated) in relation to the isokinetic strength of the knee flexors. Furthermore, peak eccentric torque is used for the calculation of the functional ratio, where the eccentric knee flexors torque is evaluated in relation to the concentric knee extensors torque. However, obtaining the functional ratio can significantly extend the duration of an already time-demanding test. Indeed, a recent systematic review [44] demonstrated no difference in association with anterior cruciate ligament and hamstring injuries between the conventional and functional ratios. Although isokinetic testing represents a valid and reliable method of assessing muscle strength at both slow and high contraction velocities, it is not without issues. Isokinetic testing necessitates the use of isokinetic dynamometers, which are costly, lack portability and demand a significant amount of time to complete their various testing protocols. Consequently, clubs with limited resources may not have access to this equipment. This has led to the search for alternative and more practical solutions, and as such, the eccentric knee flexor strength test via the use of the Nordic hamstring exercise has emerged [45]. Nordic hamstring testing enables the functional assessment of eccentric hamstring strength — a critical factor given the high prevalence of hamstrings injuries in soccer as well as the constantly increased demands of the modern game in the amount of high-speed running performed. Nordic hamstring testing is gaining popularity and currently represents the third most common method to assess strength in elite soccer based on the results of our systematic review. Its simplicity of use, the growing availability of Nordic measurement devices in elite soccer environments (e.g. Nordbord), and the ability to assess large groups of athletes in a time-efficient manner may have contributed to its rise. Furthermore, the Nordic hamstring exercise is a staple exercise in many strength and conditioning programmes in elite soccer [26, 46], and therefore no additional time for familiarisation is typically required. Finally, its well-established effectiveness in reducing the incidence of hamstring injuries [47] further validates the increased interest in assessing the amount of force produced in this exercise.

The review of the literature revealed a growing interest in the assessment of isometric and eccentric hip adductors and abductors strength over the last decade in elite soccer players. The rise in their popularity may be attributed to several factors. In addition to hamstring injuries, hip and groin injuries are also common in professional soccer, and result in long absences from training and matches [48]. The assessment of hip muscle strength, especially in the hip adductor and abductor muscle groups, plays a critical role in the clinical evaluation of groin-related issues. In fact, lower hip adduction isometric and eccentric strength values, as well as lower isometric hip adduction/abduction ratios, have been reported in athletes with groin pain [49–51]. Furthermore, hip adductors and abductors have an important function as frontal plane stabilisers, as they facilitate the prevention of excessive knee valgus during landing and cutting tasks [52]. These muscles have a significant contribution to the effective execution of COD tasks, potentially by assisting in the generation of propulsion in the lateral plane [53]. In addition, the increased availability of specialised equipment (e.g. ForceFrame, GroinBar, Kangatech KT360) in the field has led to an easier assessment of these muscles. However, the available literature suggests a lack of standardised protocols in isometric adductors and abductors strength testing. Different joint angles (hip: 0–60°, knee: 0–90°), duration of force application (3 vs 5 s), measurement devices (hand-held dynamometers, ForceFrame, GroinBar), limb engagement (unilateral vs bilateral) and outcome variables (e.g. peak force vs peak torque) have been identified in the examined literature. In view of these inconsistencies, standardisation of the overall process of assessing isometric hip adductor and abductor strength seems to be necessary.

While the aforementioned strength tests offer valuable insights into the function of specific muscle groups, they fail to provide an indicator of overall system strength. In this regard, the squat test provides a more holistic assessment of lower-body strength. The investigated literature revealed distinct squat testing methods such as the half-back squat (11 studies), back squat (eight studies) and isoinertial loading squat (three studies). However, when delving deeper into the testing protocols, a lack of clear and consistent nomenclature is evident, especially when differentiating between the half-back squat and the back squat. More specifically, in the studies that reported the “back squat” as the selected testing method, the depth of the squat varied (i.e. 90° vs thighs below parallel vs no information provided on the depth of the movement). Different squat depth has been shown to result in varying levels of muscle activation in the lower limb muscles, with greater depths leading to an increased activation of quadriceps, hamstrings and glutes muscles [54, 55]. Furthermore, individuals are able to lift heavier loads when the range of motion is shorter [56]. This can lead to inconsistent testing results and an inability to perform reliable comparisons. In addition, this discrepancy can have significant implications for the findings of this review by affecting the ranking in test frequency. In fact, better defined and standardised protocols could place either the “half-back” squat or the back squat among the three most popular testing methods in elite soccer. Overall, further standardisation of the squat test is necessary, taking into account the various 1RM calculation methods (direct assessment of 1RM vs estimation of 1RM [i.e. 6RM] vs assessment of barbell velocity using linear position transducers) and setups (barbell vs Smith machine vs Keiser) observed in our systematic review.

Finally, a lack of emphasis on upper body strength and multi-joint isometrics assessment seems to exist in elite soccer. The limited number of studies evaluating upper body strength, using exclusively the bench press test, may be possibly attributed to the specific demands of soccer, where the involvement of the upper body is minimal compared with the lower body. In contrast, similar previous work in rugby and basketball reported that the bench press test is one of the key tests in the assessment of strength [35, 36]. Isometric testing can serve as a quicker and less exhaustive alternative to dynamic testing, and both the isometric midthigh pull (IMTP) and isometric squat have been shown to be reliable options [57]. A recent survey investigating the fitness testing practices of elite male soccer practitioners identified the IMTP as the most commonly used test to assess strength [58]. Nevertheless, this systematic review identified only a single study utilising the IMTP [59], illustrating a discrepancy between research and practice. The specialised equipment required (i.e. force plates) to administer the IMTP may have rendered this test less viable in smaller clubs, accounting for the lower prevalence of the IMTP in this systematic review. In addition, isometric tests have the potential to be used in conjunction with other strength and power assessments to provide a more comprehensive picture of an athlete’s strength and power capabilities, as well as informing the training prescription; for example, with the CMJ for the assessment of a Dynamic Strength Index [60]. However, this systematic review failed to identify any studies in elite male soccer using a Dynamic Strength Index. Furthermore, the IMTP offers the ability to record multiple variables such as peak force, force at specific timepoints, rate of force development and impulse, as well as enable the identification of interlimb asymmetries. Therefore, more nuanced insights on force production can be provided. However, caution should be exercised in the use of time-dependent metrics such as rate of force development and impulse, as it has been demonstrated that their reliability is lower compared with non-time-dependent metrics, such as peak force [57, 61].

#### Power Assessment

Jump tests represent the main method for assessing power in elite soccer, with the CMJ, SJ and VJ being the most popular protocols. Jump height, measured in centimetres, was the primary outcome variable in these tests and the CMJ was by far the most commonly employed method for assessing power in elite soccer, having been featured in 99 studies. The CMJ is an easy to administer and time-efficient test that requires minimal familiarisation. Furthermore, it provides valuable insights into an athlete’s ability to utilise the stretch–shortening cycle. As hands are typically fixed on the hips during the execution of the test, this elimination of arm swing adds further standardisation to the test in assessing lower body power. A range of different equipment types, including force plates, photoelectric systems and jump mats has been employed in CMJ testing in the examined literature [62–66]; however, force plates are considered the gold-standard equipment for measuring vertical jump height [67]. In terms of calculating jump height, take-off velocity and the flight-time methods constitute the two primary methods [68]. Overall, practitioners are encouraged to use the take-off velocity method with the use of force plates [69], which are often inaccessible in the applied settings. As a result, flight time represents the most frequently used method in the calculation of jump height. However, this approach is not without its limitations. In particular, the flight-time method requires an individual to maintain the same position at take-off and landing, yet the landing position is different owing to the preparation with the ground contact mechanisms (i.e. ankle dorsiflexion and hip and knee flexion) [69]. This leads to an overestimation of the jump height. As a result, jump scores obtained using the flight-time method should not be compared with those obtained using the take-off velocity method, unless a correction equation is implemented [70]. Recently, there has been a call in the field to move beyond jump height and delve deeper into more nuanced metrics, in order to assess and report the movement strategy of the jump [71]. In this way, a more comprehensive understanding of the specific factors underlying a jump can be achieved, thereby leading to more targeted and individualised training interventions. Our literature search identified 12 additional variables, with peak power, both in absolute and relative terms, being the most frequently reported. However, similar to jump height, peak power is classified as an outcome variable that does not reveal the underlying kinetics and kinematics of the jump. Interestingly, the vast of majority of these metrics have been reported in studies conducted within the last 10 years, possibly indicating the increased availability of force plates, as well as a shift towards a more holistic assessment of jumping ability. However, given the high degree of variability found within some strategy metrics compared to jump height [72, 73], careful consideration is warranted in the selection of these.

Power is a multi-faceted concept, and as such, a single test is unlikely to provide a comprehensive assessment of power ability. This is further supported by the different types of jumps identified in this literature review. One such example is the SJ, which theoretically evaluates an athlete’s explosive ability in the absence of a stretch–shortening cycle, as no countermovement is allowed. Based on our systematic review, there is a high prevalence of SJ testing in elite soccer, with 48 studies utilising this test to assess power. The different insights provided compared to CMJ may contribute to a more comprehensive profile of power ability. Nevertheless, strict compliance with the SJ protocol (i.e. isometric hold of 2–3 s prior to the jump) is necessary, as a small-amplitude counter-movement has been shown to affect the jump height achieved [74]. In particular, the authors found that 55% of the SJ trials in their study consisted of a small-amplitude counter-movement when a gross observation was used. However, the occurrence of a small-amplitude counter-movement was increased though to 89% when the trials were analysed using force plates and to 99% when using linear position transducers. This can have significant implications in practical settings, where access to specialised equipment and resources to analyse each jump are limited. In light of these considerations, practitioners should critically evaluate the value of the information provided by the SJ. In addition, a number of studies performed the assessment of SJ under loaded conditions, which is commonly referred to as the jump squat test, using linear position transducers. In this way, an individual’s force–velocity profile and theoretical optimum power zone can be determined [75], subsequently informing targeted training interventions. Last, the VJ is another test commonly performed in elite soccer, featuring in 29 studies in this systematic review. The VJ has many similarities to the CMJ, except that an arm swing is allowed. This inclusion of the arms introduces a coordinative element to the movement and can facilitate the attainment of a higher jump height owing to the increased work output of the lower limbs that results from the use of the arm swing [76].

In recent years, the SLCMJ has garnered an increased amount of attention as a method to evaluate unilateral power in elite soccer. In fact, all 12 studies that used SLCMJ testing were conducted within the last 9 years, further highlighting the growing popularity of this test. Compared with other popular jump tests, the SLCMJ enables the assessment of power in a unilateral manner, something that can be of value given the requirement for unilateral movement competency in soccer. Moreover, the detection of interlimb asymmetries can support injury prevention and return-to-play strategies [66, 77]. Jump height, measured in centimetres, was found to be the main outcome variable obtained from the SLCMJ test. More importantly, jump height (when assessed unilaterally) has been shown to be a sensitive measure for assessing changes in performance of elite soccer players when in a fatigued state [64], encouraging the use of the test in settings where there is lack of specialised equipment such as force plates.

Interestingly, our systematic review revealed that the assessment of reactive strength ability, which represents the ability to transition rapidly from an eccentric to a concentric muscle action, does not appear to be prioritised in elite soccer players. The drop jump represents one of the most popular tests to evaluate reactive strength and provide insights into an athlete’s fast stretch–shortening cycle ability [37]. However, it was reported in only eight studies of elite soccer. In terms of outcome variables, jump height (six studies) and contact time (four studies) were the most frequently reported. The combination of these can be used to calculate the reactive strength index, which was reported in three studies and provides a measure to evaluate an individual’s reactive strength ability. Nevertheless, the fact that the reactive strength index is a ratio and is deemed as an outcome variable points to the need to examine each component separately and delve deeper into metrics that provide insights into the strategy used, such as ground contact time and leg stiffness [71].

### Normative Values for Strength and Power Tests

Normative standards can serve as an important tool in the athletic development process, enabling benchmarking and a data-informed approach to athletic development. Given the potential of strength and power to distinguish between different playing levels [16, 17], availability of normative data can provide multiple benefits to key stakeholders such as coaching and management staff. In particular, normative values for elite soccer players can support practitioners in setting training priorities and objectives, which can lead to the implementation of targeted training interventions. In addition, knowledge of the strength and power outputs of soccer players competing at the highest level can be of great value, including for practitioners working with developmental players. This can enable the reverse engineering of the strength and power development process so that players are ready to cope with the physical demands of elite soccer. Therefore, this review also provides a summary of normative values of strength and power. Owing to the large discrepancy in testing methods identified, only normative values of the most commonly implemented tests and outcome variables were reported. Overall, the biggest challenge encountered in the establishment of normative standards lies in the wide variability of testing protocols and measurement devices. Therefore, readers are referred to Tables 4–9 for more in-depth information on the values reported by each study for each test.

A variety of angular velocities, with a range from 30°/s to 300°/s, have been used in isokinetic strength testing, enabling practitioners to gain insight into muscle strength capabilities at different speeds. As the majority of isokinetic strength values were reported at 60°/s in this literature review, the mean values reported correspond to this angular velocity. The substantially smaller number of studies reporting isokinetic strength values in elite youth soccer players could possibly indicate a research area where more work needs to be performed in the future. In terms of Nordic hamstring strength, the difficulty to draw conclusions was arguably greater. More specifically, the range of mean values of peak force is large (338.2 vs 636.5 N) in the two identified studies performed in elite youth soccer players, perhaps in part as a consequence of the different equipment used (Nordic assessment device vs acceleration leg curl/extension, neuroexcellence) or the training approaches adopted by the club. In a similar manner, the variety of equipment used in jump testing such as force plates, jump mats and photoelectric cells can introduce a varying degree of measurement error during a jump assessment. In addition, readers should take into account the detailed discussion on the intricacies of jump height calculation provided in Sect. 4.1.2. The range of values observed for SJ, CMJ and VJ corroborate this observation, and as such, generalisation of these results should be avoided.

Although the normative values presented in this review can offer valuable insights to practitioners, thereby enhancing the practical utility of this work, they are subject to many limitations. Careful interpretation and application of these results is therefore recommended. Additional research is required to establish specific thresholds for each playing standard. Finally, further standardisation of data analysis is required, as it was observed that some studies reported the mean value of the trials performed. Currently, it may be advised to determine club specific standards and compare players against this, thus accounting for differences in test equipment, methodology and the adopted culture and philosophy of training and testing [78].

### Reliability Data

Reliability is an important concept in the overall testing process, especially in high-performance sport where success depends on marginal differences. The use of reliable tests and outcome variables can ensure that the data collected reflects an athlete’s true capacity, therefore guiding effective decision making. Nevertheless, our systematic review revealed that a relatively small number of studies reported reliability data for strength and power tests (15 and 34 studies, respectively), impairing confidence in the interpretation of test results and performance changes. This finding highlights the need to generate awareness of the utility of these metrics within the prescription and reporting of testing. It is of paramount importance for practitioners to establish their own reliability measures within their specific contexts, as the characteristics of each setting and athlete sample are unique.

Intra-day reliability was the most common type of reliability. The predominance of intra-day reliability could be attributed to the inherent complexity of conducting between-day reliability studies in elite sports. More specifically, when aiming to undertake inter-day reliability assessments, the second assessment is usually performed within 3–7 days of the first [79], which is not always feasible because of demanding training and competition schedules. Determining the between-day variability, though, can promote a more holistic and evidence-informed interpretation of performance changes, as the between-day variability is not typically the same as within-day data because biological variation is also factored in. In fact, our review revealed generally higher values for the intra-day reliability of the CMJ and SJ height compared with the inter-day reliability values reported in one study [80]. A similar observation was made in Nordic hamstring strength testing, where intra-day reliability values were considerably higher than inter-day reliability values. To address this, more ecological approaches to between-day reliability testing have been recently introduced in elite soccer [80] and rugby union [72], by integrating the reliability testing within the microcycle, where normal training is undertaken in the days prior to the re-assessment.

In terms of reliability metrics, the ICC was the most frequently reported. The ICC is a measure of relative reliability, which is the extent to which an individual maintains their ranking over the course of repeated trials. Although what is an acceptable ICC value can be debatable, it is generally embraced that the ICC ≥ 0.75 is considered as “good” reliability, with an ICC ≥ 0.90 considered as “excellent” [81]. Nonetheless, the ICC is influenced by group homogeneity and does not provide any information on the variation between efforts of an individual. Therefore, it is crucial for absolute reliability to also be established. Based on our findings, the CV and SEM were the most common metrics to evaluate absolute reliability. The CV indicates the relative dispersion of the data points around the mean by expressing the SD as a percentage of the mean, while the SEM provides an index of the precision of the measurement by estimating the range in which the population’s true score is expected to lie, within a defined level of confidence. In addition, these measures are more relevant to practice, as they are used for the assessment of sensitivity. Although the scientific community seems to broadly recognise the value of ≤ 10% as an acceptable threshold, this threshold appears to be rather arbitrary and a more nuanced and context-specific interpretation is required [82]. The paper by Mercer et al. [73] demonstrated that although certain CMJ variables produce a CV > 10%, they are still sensitive to training changes, justifying their use in practice. Readers are directed to this article to gain insights on how to determine the signal-to-noise ratio in an ecologically valid and non-disruptive to the training process manner, with their own athletes.

Regarding strength testing, the smaller number of studies reporting reliability values means that conclusions should be drawn with caution. The half-back squat seems to possess high levels of intra-day (ICC [0.94–0.97], CV [1.8–3.1], SEM [1.71]) and inter-day reliability (ICC [0.99], CV [1.8], SEM [2]). Additionally, the Nordic hamstring strength test appears to have high intra-day relative reliability levels (ICC [0.97–0.99]) in conjunction with a small CV value (1.0–3.2). In terms of inter-day reliability of the Nordic hamstring strength test, the only study performed demonstrated moderate levels of relative reliability, but with a CV value below 10%. Regarding power testing, the reported ICC ranges in CMJ (0.80–0.99), SJ (0.75–0.99) and SLCMJ (0.74–0.99) height, coupled with their CV (CMJ [1.8–15], SJ [2.1–13], SLCMJ [1.9–9.6]) and SEM (CMJ [0.6–1.4], SJ [0.6], SLCMJ [0.3–1]) values identified in this systematic review, confirm their high level of reliability. The increased availability of force plates in elite soccer will warrant the determination of reliability, particularly between days, in metrics other than jump height, representing an area where future research in elite soccer should focus. Although the current reliability data are generally robust, practitioners should still validate these measures within their specific context to ensure the accuracy and applicability of the data.

Finally, only a very limited number of studies reported the MDC. Minimal detectable change, calculated from the SEM, illustrates the minimal amount of change in performance required to be confident that the change can be considered as real at a predetermined probability level (usually 90% or 95%). This may raise the need to further determine MDC of strength and power tests in the context of elite soccer, as this will allow practitioners to identify normal variations or true changes in performance. However, it should be acknowledged that such a high confidence threshold may not be suitable for high-performance settings where a high level of physical performance has already been established and training interventions can therefore only elicit a certain degree of positive adaptations. This can lead to tiny but significant positive changes being labelled as “noise”, resulting in the discontinuation of certain training interventions that are actually working.

### Limitations

Although this systematic review provided a comprehensive picture of strength and power testing in elite soccer, there are several limitations that should be acknowledged. To begin with, as Boullosa et al. [83] indicate, the conclusions of a systematic review can be influenced by the inclusion or exclusion of a few studies. In this sense, the terms “elite” and “professional” are often used interchangeably across the literature. It is likely, though, that these terms may be used differently in different geographic regions and leagues. This can be considered as a limitation, as well as a reflection of the existing soccer literature, highlighting the need for a standardised terminology for “elite” and “professional”. The large variability in equipment is another challenge in the establishment of normative standards, complicating the direct translation of these findings into practice. In addition, because of the heterogeneity of testing methods identified in the present literature review, it was not possible to carry out a meta-analysis. Last, in strength testing, a substantial lower number of studies reporting normative values was available. This discrepancy may interfere with the ability to perform reliable comparisons between men and young elite soccer players.

### Directions for Future Research

This systematic review has identified several areas that require further investigation. There is a need to standardise several aspects of strength and power testing to improve the comparability and the application of the results. This includes standardising the definitions, such as the distinction between the half-back squat and the back squat, and standardising procedures for certain tests, such as the isometric adductor strength test, the Nordic hamstring test and the CMJ. Future research should focus on establishing a hybrid testing framework that incorporates standardised “core” tests for benchmarking and large-scale comparisons, while allowing practitioners to introduce additional context-specific tests tailored to the unique dynamics of their settings. A scarcity of robust reliability data is evident in elite soccer. Practitioners need to establish their own reliability measures, and subsequently the sensitivity of those, within their specific contexts, to enhance confidence in assessing performance changes and reducing reliance on published reliability thresholds. This will assist in determining any of those that do not offer any particular value in decision making, removing any redundant processes and data. A standardised data analysis process should be also adopted, as there is no consensus on the optimal approach to analyse strength and power testing results (i.e. best trial vs average of trials). Future studies should therefore examine the ramifications of each approach. Last, future studies should investigate the most effective methods of reporting the testing results to the key stakeholders to enhance the impact of testing in the training process. These studies have the potential to reshape the strength and power assessment procedures in elite soccer, enabling more robust practices and informed practices.

## Conclusions

**Fig. 2:**
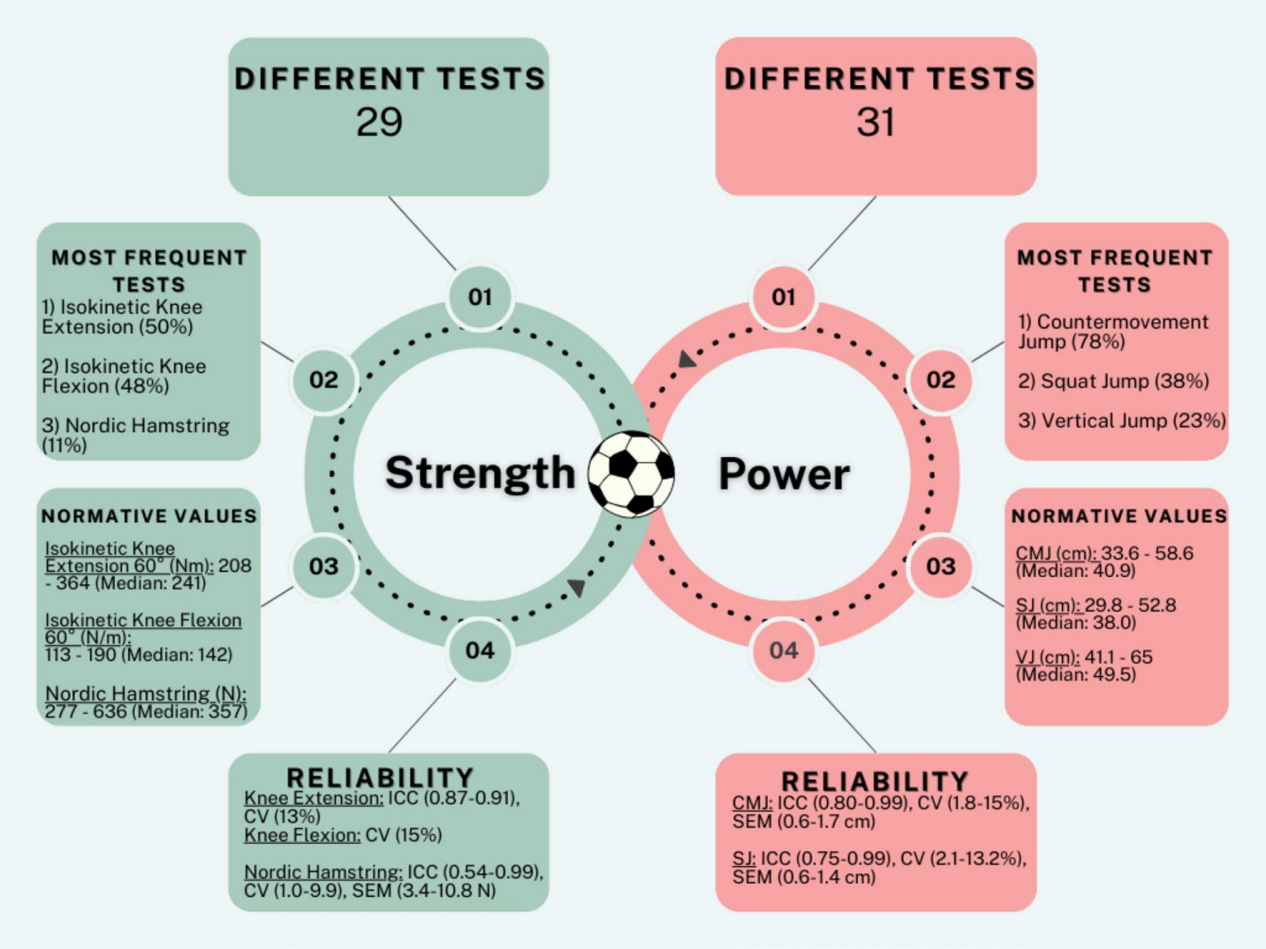
Strength and power testing in elite male soccer. *CMV* countermovement jump, *CV* coefficient of variation, *ICC* intraclass correlation coefficient, *SEM* standard error of measurement, *SJ* squat jump, *VJ* vertical jump

This systematic review, as illustrated in the infographic in Fig. 2, provides a comprehensive overview of the tests and outcome variables used to assess strength and power in elite male soccer. The wide variety of different tests employed combined with the multitude of different outcome variables indicates the lack of a consensus in strength and power testing in elite soccer. This may arise from the diverse training needs of each specific setting, as well as the different testing philosophies across cultures. In terms of frequency, isokinetic knee (extensors and flexors) strength testing and CMJ were the most administered strength and power tests, respectively. The normative values provided for these tests enhance the practicality of this review. However, the application of these normative values warrants careful consideration, as different testing protocols and instruments have been utilised. Future research should focus on the development of a hybrid testing approach to strength and power testing, combining standardised tests for benchmarking purposes, while allowing for flexible testing selection based on the unique requirements of each specific context to enable a holistic profiling of strength and power.

## Supplementary Information

Below is the link to the electronic supplementary material.

Supplementary file1 (DOCX 422 KB)

## Declarations

### Availability of Data and Material

The dataset generated and analysed during the current study are available from the corresponding author on reasonable request.
