# Supplementary material for: Assessment of Strength and Power Capacities in Elite Male Soccer: A Systematic Review of Test Protocols Used in Practice and Research
Source: Sports Med. 2024 Jul 18;54(10):2607–44. doi: 10.1007/s40279-024-02071-8 (PMC11467003; doi:10.1007/s40279-024-02071-8)
Supplement: Supplementary file 1 — Supplementary file1 (DOCX 422 KB) [file 40279_2024_2071_MOESM1_ESM.docx]

**Title:** Assessment of Strength and Power Capacities in Elite Male Soccer: A Systematic Review of Test Protocols used in Practice and Research

**Short Title:** Strength and Power Testing in Elite Male Soccer

**Authors:** Nikolaos D. Asimakidis^1,2^, Irvin N. Mukandi^1,2^, Marco Beato^3^, Chris Bishop^1^, Anthony N. Turner^1^

^1^ Faculty of Science and Technology, London Sport Institute, Middlesex University, London, UK

^2^ Performance Department, Ipswich Town Football Club, Ipswich, UK
^3^ School of Health and Sports Sciences, University of Suffolk, Ipswich, UK

**Corresponding author**: Nikolaos D. Asimakidis

n.asimakidis@gmail.com

**Supplementary Table S1** Questions used from the modified Downs and Black [34] checklist to evaluate methodological quality of the included articles

| Question no. | Question |
| --- | --- |
|  | **Reporting** |
| 1. | Is the hypothesis/aim/objective of the study clearly described? |
| 2. | Are the main outcomes to be measured clearly described in the methods section? |
| 3. | Are the characteristics of the participants included in the study clearly described?  *Source defined, with characteristics included |
| 4. | Are the interventions/testing procedures in the study clearly described? |
| 6. | Are the main findings of the study clearly described? |
| 7. | Does the study provide estimates of the random variability in the data for main outcomes? |
| 10. | Have actual probability values been reported (e.g., 0.0035 rather than < 0.05) for the main outcomes except where the probability value is < 0.001?  *Exact correlation (r) and significance (p) values provided. The reporting of effect sizes (ESs) was deemed acceptable. |
|  | **External validity** |
| 11. | Were the subjects asked to participate in the study representative of the entire population from which they were recruited? |
|  | **Internal validity bias** |
| 16. | If any of the results of the study were based on “data dredging”, was this made clear? |
| 18. | Were the statistical tests used to assess the main outcomes appropriate? |
| 20. | Were the main outcome measures used accurate (reliable)? |

**Supplementary Table S2** Assessment of methodological quality [34]

| **Author** | **Question number** | | | | | | | | | | | **Total score** |
| --- | --- | --- | --- | --- | --- | --- | --- | --- | --- | --- | --- | --- |
|  | **1** | **2** | **3** | **4** | **6** | **7** | **10** | **11** | **16** | **18** | **20** |  |
| Maestroni et al. [66] | 1 | 1 | 1 | 1 | 1 | 1 | 1 | 0 | 1 | 1 | 1 | 10 |
| Espada et al. [84] | 1 | 1 | 1 | 1 | 1 | 1 | 1 | 0 | 1 | 1 | 1 | 10 |
| Cossich et al. [85] | 1 | 1 | 1 | 1 | 1 | 1 | 1 | 0 | 1 | 1 | 1 | 10 |
| Byrkjedal et al. [86] | 1 | 1 | 1 | 1 | 1 | 1 | 1 | 0 | 1 | 1 | 1 | 10 |
| Bishop et al. [64] | 1 | 1 | 1 | 1 | 1 | 1 | 1 | 0 | 1 | 1 | 1 | 10 |
| Wezenbeek et al. [87] | 1 | 0 | 1 | 1 | 1 | 1 | 1 | 0 | 1 | 1 | 1 | 9 |
| Guerra et al. [88] | 1 | 0 | 1 | 1 | 1 | 1 | 1 | 0 | 1 | 1 | 1 | 9 |
| Papadakis et al. [89] | 1 | 1 | 1 | 1 | 1 | 1 | 1 | 0 | 1 | 1 | 1 | 10 |
| Lahti et al. [90] | 1 | 1 | 1 | 1 | 1 | 1 | 1 | 0 | 1 | 1 | 1 | 10 |
| Bongiovanni et al. [91] | 1 | 0 | 1 | 1 | 1 | 1 | 1 | 0 | 1 | 1 | 1 | 9 |
| Papla et al. [92] | 1 | 0 | 1 | 1 | 1 | 1 | 0 | 0 | 1 | 1 | 1 | 8 |
| Boraczyński et al. [93] | 1 | 1 | 1 | 1 | 1 | 1 | 1 | 0 | 1 | 1 | 1 | 10 |
| Misjuk and Rannama [94] | 1 | 1 | 1 | 1 | 1 | 1 | 1 | 0 | 0 | 1 | 1 | 9 |
| Cadu et al. [95] | 1 | 1 | 1 | 1 | 1 | 1 | 1 | 0 | 0 | 1 | 1 | 9 |
| Bishop et al. [77] | 1 | 1 | 1 | 1 | 1 | 1 | 1 | 1 | 0 | 1 | 1 | 10 |
| Keiner et al. [15] | 1 | 1 | 1 | 1 | 1 | 1 | 1 | 0 | 0 | 1 | 1 | 9 |
| Schons et al. [96] | 1 | 1 | 1 | 1 | 1 | 1 | 0 | 0 | 1 | 1 | 1 | 9 |
| Freitas et al. [97] | 1 | 1 | 1 | 1 | 1 | 1 | 1 | 0 | 1 | 1 | 1 | 10 |
| Arregui-Martin et al. [98] | 1 | 0 | 1 | 0 | 1 | 1 | 1 | 0 | 1 | 1 | 1 | 8 |
| Leporace et al. [99] | 1 | 1 | 1 | 1 | 1 | 1 | 1 | 0 | 1 | 1 | 1 | 10 |
| Querido and Clemente [100] | 1 | 1 | 1 | 1 | 1 | 1 | 1 | 0 | 1 | 1 | 1 | 10 |
| Shalaj et al. [101] | 1 | 1 | 1 | 1 | 1 | 1 | 1 | 0 | 1 | 1 | 1 | 10 |
| Manoel et al. [102] | 1 | 1 | 1 | 1 | 1 | 1 | 1 | 0 | 1 | 1 | 1 | 10 |
| Krespi et al. [103] | 1 | 0 | 0 | 1 | 1 | 1 | 1 | 0 | 1 | 1 | 1 | 8 |
| Stern et al. [14] | 1 | 1 | 1 | 1 | 1 | 1 | 1 | 0 | 1 | 1 | 1 | 10 |
| Moreno-Pérez et al. [104] | 1 | 1 | 1 | 1 | 1 | 1 | 1 | 0 | 1 | 1 | 1 | 10 |
| Correia et al. [105] | 1 | 1 | 1 | 1 | 1 | 1 | 1 | 0 | 1 | 1 | 1 | 10 |
| Ribeiro-Alvares et al. [106] | 1 | 1 | 1 | 1 | 1 | 1 | 1 | 0 | 1 | 1 | 1 | 10 |
| Suarez-Arrones et al. [107] | 1 | 1 | 1 | 1 | 1 | 1 | 1 | 0 | 1 | 1 | 1 | 10 |
| Van Klij et al. [108] | 1 | 0 | 1 | 1 | 1 | 1 | 1 | 0 | 1 | 1 | 1 | 9 |
| Ribeiro et al. [109] | 1 | 1 | 1 | 1 | 1 | 1 | 1 | 0 | 1 | 1 | 1 | 10 |
| Lockie et al. [110] | 1 | 1 | 1 | 1 | 1 | 1 | 1 | 0 | 1 | 1 | 1 | 10 |
| Ishøi et al. [111] | 1 | 1 | 1 | 1 | 1 | 1 | 1 | 0 | 1 | 1 | 1 | 10 |
| Bishop et al. [77] | 1 | 1 | 1 | 1 | 1 | 1 | 1 | 0 | 1 | 1 | 1 | 10 |
| Moreno-Pérez et al. [112] | 1 | 1 | 1 | 1 | 1 | 1 | 1 | 0 | 1 | 1 | 1 | 10 |
| Dolci et al. [113] | 1 | 1 | 1 | 1 | 1 | 1 | 1 | 0 | 1 | 1 | 1 | 10 |
| Izovska et al. [43] | 1 | 1 | 1 | 1 | 1 | 1 | 1 | 0 | 1 | 1 | 1 | 10 |
| Nielsen et al. [114] | 1 | 1 | 1 | 1 | 1 | 1 | 1 | 0 | 1 | 1 | 1 | 10 |
| Silva et al. [115] | 1 | 1 | 1 | 1 | 1 | 0 | 0 | 0 | 1 | 1 | 1 | 8 |
| Light et al. [116] | 1 | 1 | 1 | 1 | 1 | 1 | 1 | 0 | 1 | 1 | 1 | 10 |
| Saccà et al. [117] | 1 | 1 | 0 | 0 | 1 | 1 | 1 | 0 | 1 | 1 | 1 | 8 |
| Enes et al. [118] | 1 | 1 | 1 | 1 | 1 | 1 | 1 | 0 | 1 | 1 | 1 | 10 |
| Ben Brahim et al. [119] | 1 | 1 | 1 | 1 | 1 | 1 | 1 | 0 | 1 | 1 | 1 | 10 |
| Bishop et al. [63] | 1 | 1 | 1 | 1 | 1 | 1 | 1 | 0 | 1 | 1 | 1 | 10 |
| Rodrigues Júnior et al. [120] | 1 | 1 | 1 | 1 | 1 | 1 | 1 | 0 | 1 | 1 | 1 | 10 |
| Capaverde et al. [121] | 1 | 1 | 1 | 1 | 1 | 1 | 1 | 0 | 0 | 1 | 1 | 9 |
| Ribeiro-Alvares et al. [122] | 1 | 1 | 1 | 1 | 1 | 1 | 1 | 0 | 1 | 1 | 1 | 10 |
| Scoz et al. [123] | 1 | 1 | 1 | 1 | 1 | 1 | 0 | 0 | 1 | 1 | 1 | 9 |
| Bourne et al. [124] | 1 | 1 | 1 | 1 | 1 | 1 | 1 | 0 | 1 | 1 | 1 | 10 |
| Ocarino et al. [125] | 1 | 1 | 1 | 1 | 1 | 1 | 1 | 0 | 1 | 1 | 1 | 10 |
| Read et al. [126] | 1 | 1 | 1 | 1 | 1 | 1 | 0 | 0 | 1 | 1 | 1 | 9 |
| Beato et al. [18] | 1 | 1 | 1 | 1 | 1 | 1 | 1 | 0 | 1 | 1 | 1 | 10 |
| Cardoso De Araújo et al. [127] | 1 | 1 | 1 | 1 | 1 | 1 | 1 | 0 | 1 | 1 | 0 | 9 |
| Loturco et al. [128] | 1 | 1 | 1 | 1 | 1 | 1 | 1 | 0 | 1 | 1 | 1 | 10 |
| Papla et al. [129] | 1 | 1 | 1 | 1 | 1 | 1 | 1 | 0 | 1 | 1 | 1 | 10 |
| Loturco et al. [130] | 1 | 1 | 1 | 1 | 1 | 1 | 1 | 0 | 1 | 1 | 1 | 10 |
| Grazioli et al. [131] | 1 | 1 | 1 | 1 | 1 | 1 | 1 | 0 | 1 | 1 | 1 | 10 |
| Śliwowski et al. [132] | 1 | 1 | 1 | 1 | 1 | 1 | 1 | 0 | 1 | 1 | 1 | 10 |
| Papadakis et al. [65] | 1 | 1 | 1 | 1 | 1 | 1 | 1 | 0 | 1 | 1 | 1 | 10 |
| Eustace et al. [133] | 1 | 1 | 1 | 1 | 1 | 1 | 1 | 0 | 1 | 1 | 1 | 10 |
| Hoppe et al. [17] | 1 | 1 | 1 | 1 | 1 | 1 | 0 | 0 | 1 | 1 | 0 | 8 |
| Arcos et al. [134] | 1 | 1 | 1 | 1 | 1 | 1 | 1 | 0 | 1 | 1 | 1 | 10 |
| Boraczyński et al. [135] | 1 | 1 | 1 | 1 | 1 | 1 | 1 | 0 | 1 | 1 | 1 | 10 |
| Bianchi et al. [136] | 1 | 1 | 1 | 1 | 1 | 1 | 1 | 0 | 1 | 1 | 1 | 10 |
| Michaelides et al. [137] | 1 | 1 | 1 | 1 | 1 | 1 | 1 | 0 | 1 | 1 | 1 | 10 |
| Saidi et al. [138] | 1 | 1 | 1 | 1 | 1 | 1 | 1 | 0 | 1 | 1 | 0 | 9 |
| Loturco et al. [139] | 1 | 1 | 1 | 1 | 1 | 1 | 1 | 0 | 1 | 1 | 1 | 10 |
| Northeast et al. [59] | 1 | 0 | 1 | 1 | 1 | 1 | 0 | 0 | 1 | 1 | 1 | 8 |
| Van Dyk et al. [140] | 1 | 0 | 1 | 1 | 1 | 1 | 1 | 0 | 1 | 1 | 1 | 9 |
| Moreno-Pérez et al. [141] | 1 | 1 | 1 | 1 | 1 | 1 | 1 | 0 | 1 | 1 | 1 | 10 |
| López-Valenciano et al. [142] | 1 | 1 | 1 | 1 | 1 | 1 | 0 | 0 | 1 | 1 | 1 | 9 |
| Enright et al. [80] | 1 | 1 | 1 | 1 | 1 | 1 | 1 | 0 | 1 | 1 | 1 | 10 |
| Rago et al. [143] | 1 | 1 | 1 | 0 | 1 | 1 | 1 | 0 | 1 | 1 | 1 | 9 |
| Loturco et al. [144] | 1 | 1 | 1 | 1 | 1 | 1 | 0 | 0 | 1 | 1 | 1 | 9 |
| Mosler et al. [145] | 1 | 1 | 1 | 1 | 1 | 1 | 1 | 0 | 1 | 1 | 1 | 10 |
| Mosler et al. [146] | 1 | 1 | 1 | 1 | 1 | 1 | 1 | 0 | 1 | 1 | 1 | 10 |
| Los Arcos et al. [147] | 1 | 1 | 1 | 1 | 1 | 1 | 1 | 0 | 1 | 1 | 1 | 10 |
| Almeida et al. [148] | 1 | 0 | 1 | 1 | 0 | 1 | 1 | 0 | 1 | 1 | 1 | 8 |
| Coratella et al. [149] | 1 | 1 | 1 | 1 | 1 | 1 | 1 | 0 | 1 | 1 | 1 | 10 |
| Suarez-Arrones et al. [150] | 1 | 1 | 1 | 1 | 1 | 0 | 1 | 0 | 1 | 1 | 1 | 9 |
| Gil et al. [151] | 1 | 1 | 1 | 1 | 1 | 1 | 1 | 0 | 1 | 1 | 1 | 10 |
| Śliwowski et al. [152] | 1 | 1 | 1 | 1 | 1 | 1 | 1 | 0 | 1 | 1 | 1 | 10 |
| Van Dyk et al. [153] | 1 | 1 | 1 | 1 | 1 | 1 | 1 | 0 | 1 | 1 | 1 | 10 |
| Buśko et al. [154] | 1 | 1 | 1 | 1 | 1 | 1 | 1 | 0 | 1 | 1 | 1 | 10 |
| Bakken et al. [155] | 1 | 1 | 1 | 1 | 1 | 1 | 1 | 0 | 1 | 1 | 1 | 10 |
| Lee et al. [156] | 1 | 1 | 1 | 1 | 1 | 1 | 1 | 0 | 1 | 1 | 1 | 10 |
| Haugen [157] | 1 | 1 | 0 | 1 | 1 | 1 | 0 | 0 | 1 | 1 | 1 | 8 |
| Murtagh et al. [158] | 1 | 0 | 1 | 1 | 1 | 1 | 1 | 0 | 1 | 1 | 1 | 9 |
| Van Dyk et al. [42] | 1 | 1 | 1 | 1 | 1 | 1 | 1 | 0 | 1 | 1 | 1 | 10 |
| Kobal et al. [159] | 1 | 1 | 1 | 1 | 1 | 1 | 1 | 0 | 1 | 1 | 1 | 10 |
| Otero-Esquina et al. [13] | 1 | 0 | 1 | 1 | 1 | 1 | 1 | 0 | 1 | 1 | 1 | 9 |
| Śliwowski et al. [160] | 1 | 1 | 1 | 1 | 1 | 1 | 1 | 0 | 1 | 1 | 1 | 10 |
| Abade et al. [161] | 1 | 1 | 1 | 1 | 1 | 1 | 1 | 0 | 1 | 1 | 1 | 10 |
| Requena et al. [162] | 1 | 1 | 1 | 0 | 1 | 1 | 0 | 0 | 1 | 1 | 1 | 8 |
| Light and Thorborg [163] | 1 | 1 | 1 | 1 | 1 | 1 | 1 | 0 | 1 | 1 | 1 | 10 |
| Loturco et al. [164] | 1 | 1 | 1 | 1 | 1 | 1 | 1 | 0 | 1 | 1 | 1 | 10 |
| Mosler et al. [165] | 1 | 1 | 1 | 1 | 1 | 1 | 1 | 0 | 1 | 1 | 1 | 10 |
| Pareja-Blanco et al. [166] | 1 | 1 | 1 | 1 | 1 | 1 | 1 | 0 | 1 | 1 | 1 | 10 |
| Krommes et al. [167] | 1 | 1 | 1 | 1 | 1 | 1 | 0 | 0 | 1 | 0 | 1 | 8 |
| Yanci and Los Arcos [168] | 1 | 1 | 1 | 1 | 1 | 1 | 1 | 0 | 1 | 1 | 1 | 10 |
| Timmins et al. [45] | 1 | 1 | 1 | 1 | 1 | 1 | 1 | 0 | 1 | 1 | 1 | 10 |
| Belhaj et al. [169] | 1 | 1 | 1 | 1 | 1 | 1 | 1 | 0 | 1 | 1 | 1 | 10 |
| Van Dyk et al. [170] | 1 | 0 | 1 | 1 | 1 | 1 | 1 | 0 | 1 | 1 | 1 | 9 |
| De Hoyo et al. [12] | 1 | 1 | 1 | 1 | 1 | 1 | 1 | 0 | 1 | 1 | 1 | 10 |
| Fessi et al. [171] | 1 | 0 | 1 | 1 | 1 | 1 | 0 | 0 | 1 | 1 | 1 | 8 |
| Kobal et al. [16] | 1 | 1 | 1 | 1 | 1 | 1 | 1 | 0 | 1 | 1 | 1 | 10 |
| Spineti et al. [172] | 1 | 1 | 1 | 1 | 1 | 1 | 1 | 0 | 1 | 1 | 1 | 10 |
| Carvalho et al. [173] | 1 | 1 | 1 | 1 | 1 | 1 | 1 | 0 | 1 | 1 | 1 | 10 |
| Rey et al. [174] | 1 | 1 | 1 | 0 | 1 | 1 | 1 | 0 | 1 | 1 | 1 | 9 |
| Martinez-Santos et al. [62] | 1 | 1 | 1 | 1 | 1 | 1 | 1 | 0 | 1 | 1 | 1 | 10 |
| Loturco et al. [175] | 1 | 1 | 1 | 1 | 1 | 1 | 1 | 0 | 1 | 1 | 1 | 10 |
| Noon et al. [176] | 1 | 0 | 1 | 1 | 1 | 1 | 0 | 0 | 1 | 1 | 1 | 8 |
| Enright et al. [177] | 1 | 0 | 1 | 1 | 1 | 1 | 1 | 0 | 1 | 1 | 1 | 9 |
| Ruas et al. [178] | 1 | 0 | 1 | 1 | 1 | 1 | 0 | 0 | 1 | 1 | 1 | 8 |
| Loturco et al. [75] | 1 | 1 | 1 | 1 | 1 | 1 | 1 | 0 | 1 | 1 | 1 | 10 |
| Tsiokanos et al. [179] | 1 | 1 | 1 | 1 | 1 | 1 | 0 | 0 | 1 | 1 | 1 | 9 |
| Pareja-Blanco et al. [180] | 1 | 1 | 1 | 1 | 1 | 1 | 1 | 0 | 1 | 1 | 1 | 10 |
| Bogdanis and Kalapotharakos [181] | 1 | 1 | 1 | 1 | 1 | 1 | 1 | 0 | 1 | 1 | 1 | 10 |
| Gil et al. [182] | 1 | 1 | 1 | 1 | 1 | 1 | 1 | 0 | 1 | 1 | 1 | 10 |
| Loturco et al. [183] | 1 | 1 | 1 | 1 | 1 | 1 | 1 | 0 | 1 | 1 | 1 | 10 |
| Ardern et al. [184] | 1 | 1 | 1 | 1 | 1 | 1 | 0 | 0 | 1 | 1 | 1 | 9 |
| Loturco et al. [185] | 1 | 0 | 1 | 1 | 1 | 1 | 1 | 0 | 1 | 1 | 1 | 9 |
| Ruas et al. [186] | 1 | 1 | 1 | 1 | 1 | 1 | 1 | 0 | 1 | 1 | 1 | 10 |
| Arcos et al. [187] | 1 | 1 | 1 | 1 | 1 | 1 | 1 | 0 | 1 | 1 | 1 | 10 |
| Booysen et al. [188] | 1 | 1 | 1 | 1 | 1 | 1 | 1 | 0 | 1 | 1 | 1 | 10 |
| Owen et al. [189] | 1 | 1 | 1 | 1 | 1 | 1 | 1 | 0 | 1 | 1 | 0 | 9 |
| Tol et al. [190] | 1 | 0 | 0 | 1 | 1 | 1 | 1 | 0 | 1 | 1 | 1 | 8 |
| Edholm et al. [191] | 1 | 1 | 1 | 1 | 1 | 1 | 0 | 0 | 1 | 1 | 1 | 9 |
| Brocherie et al. [192] | 1 | 1 | 1 | 1 | 1 | 1 | 1 | 0 | 1 | 1 | 1 | 10 |
| Haddad et al. [193] | 1 | 1 | 1 | 1 | 1 | 1 | 1 | 0 | 1 | 1 | 1 | 10 |
| Arcos et al. [194] | 1 | 1 | 1 | 1 | 1 | 1 | 1 | 0 | 1 | 1 | 1 | 10 |
| Koundourakis et al. [195] | 1 | 1 | 1 | 0 | 1 | 1 | 1 | 0 | 1 | 1 | 1 | 9 |
| Koundourakis et al. [196] | 1 | 1 | 1 | 1 | 1 | 1 | 1 | 0 | 1 | 1 | 1 | 10 |
| Portella et al. [197] | 1 | 1 | 1 | 1 | 1 | 1 | 1 | 0 | 1 | 1 | 1 | 10 |
| Thorborg et al. [50] | 1 | 1 | 1 | 1 | 1 | 1 | 1 | 0 | 1 | 1 | 1 | 10 |
| Requena et al. [198] | 1 | 1 | 1 | 1 | 1 | 1 | 1 | 0 | 1 | 1 | 1 | 10 |
| Haugen et al. [199] | 1 | 1 | 1 | 1 | 1 | 1 | 1 | 0 | 1 | 1 | 1 | 10 |
| Rebelo et al. [200] | 1 | 1 | 1 | 1 | 1 | 1 | 1 | 0 | 1 | 1 | 1 | 10 |
| Menzel et al. [201] | 1 | 0 | 1 | 1 | 1 | 1 | 1 | 0 | 1 | 1 | 1 | 9 |
| Silva et al. [202] | 1 | 1 | 1 | 1 | 1 | 1 | 1 | 0 | 1 | 1 | 1 | 10 |
| Loturco et al. [203] | 1 | 1 | 1 | 1 | 1 | 1 | 0 | 0 | 1 | 1 | 1 | 9 |
| Castagna and Castellini [204] | 1 | 1 | 1 | 1 | 1 | 1 | 1 | 0 | 1 | 1 | 1 | 10 |
| Lago-Ballesteros [205] | 1 | 1 | 1 | 1 | 1 | 1 | 0 | 0 | 1 | 1 | 1 | 9 |
| Daneshjoo et al. [206] | 1 | 1 | 1 | 1 | 1 | 1 | 1 | 0 | 1 | 1 | 1 | 10 |
| Boone et al. [207] | 1 | 1 | 1 | 1 | 1 | 1 | 1 | 0 | 1 | 1 | 1 | 10 |
| Chaouachi et al. [208] | 1 | 0 | 1 | 1 | 1 | 0 | 1 | 0 | 1 | 1 | 1 | 8 |
| Daneshjoo et al. [209] | 1 | 1 | 1 | 1 | 1 | 1 | 1 | 0 | 1 | 1 | 1 | 10 |
| Whiteley et al. [210] | 1 | 1 | 0 | 1 | 1 | 1 | 1 | 0 | 1 | 0 | 1 | 8 |
| Greco et al. [211] | 1 | 1 | 1 | 1 | 1 | 1 | 1 | 0 | 1 | 1 | 1 | 10 |
| Requena et al. [212] | 1 | 0 | 0 | 1 | 1 | 1 | 0 | 0 | 1 | 1 | 1 | 7 |
| Silva et al. [213] | 1 | 1 | 1 | 1 | 1 | 1 | 0 | 0 | 1 | 1 | 1 | 9 |
| Jovanovic et al. [214] | 1 | 0 | 1 | 0 | 1 | 0 | 0 | 0 | 0 | 0 | 1 | 4 |
| Helgerud et al. [215] | 1 | 1 | 1 | 1 | 1 | 1 | 1 | 0 | 1 | 1 | 1 | 10 |
| Rønnestad et al. [216] | 1 | 1 | 1 | 1 | 1 | 1 | 0 | 0 | 1 | 1 | 1 | 9 |
| Bogdanis et al. [217] | 1 | 1 | 1 | 1 | 1 | 1 | 1 | 0 | 1 | 1 | 1 | 10 |
| Faude et al. [218] | 1 | 1 | 1 | 1 | 1 | 1 | 1 | 0 | 1 | 1 | 1 | 10 |
| Zebis et al. [219] | 1 | 1 | 1 | 1 | 1 | 1 | 1 | 0 | 1 | 1 | 1 | 10 |
| Cotte and Chatard [220] | 1 | 1 | 1 | 1 | 1 | 1 | 0 | 0 | 1 | 1 | 1 | 9 |
| Henderson et al. [221] | 1 | 0 | 1 | 0 | 1 | 1 | 0 | 0 | 1 | 1 | 1 | 7 |
| Wong et al. [222] | 1 | 1 | 1 | 1 | 1 | 1 | 0 | 0 | 1 | 1 | 1 | 9 |
| López-Segovia et al. [223] | 1 | 0 | 1 | 1 | 1 | 1 | 1 | 0 | 1 | 1 | 1 | 9 |
| Till and Cooke [224] | 1 | 1 | 1 | 1 | 1 | 1 | 0 | 0 | 1 | 1 | 1 | 9 |
| Mujika et al. [225] | 1 | 1 | 1 | 1 | 1 | 1 | 0 | 0 |  | 1 | 1 | 8 |
| Mujika et al. [226] | 1 | 1 | 1 | 1 | 1 | 1 | 1 | 0 | 1 | 1 | 1 | 10 |
| Sporis et al. [227] | 1 | 0 | 1 | 1 | 1 | 1 | 0 | 0 | 1 | 1 | 1 | 8 |
| Bravo et al. [228] | 1 | 1 | 1 | 1 | 1 | 1 | 1 | 0 | 1 | 1 | 1 | 10 |
| Croisier et al. [20] | 1 | 1 | 1 | 1 | 1 | 1 | 0 | 0 | 1 | 1 | 1 | 9 |
| Lehance et al. [21] | 1 | 1 | 1 | 1 | 1 | 1 | 0 | 0 | 1 | 1 | 1 | 9 |
| Hoshikawa et al. [229] | 1 | 1 | 1 | 1 | 1 | 1 | 1 | 0 | 1 | 1 | 1 | 10 |
| Clark et al. [230] | 1 | 1 | 1 | 1 | 1 | 1 | 1 | 0 | 1 | 1 | 1 | 10 |
| Ronnestad et al. [231] | 1 | 1 | 1 | 1 | 1 | 1 | 1 | 0 | 1 | 1 | 1 | 10 |
| Chamari et al. [232] | 1 | 1 | 0 | 1 | 1 | 1 | 1 | 0 | 1 | 1 | 1 | 9 |
| Cressey et al. [233] | 1 | 1 | 0 | 1 | 1 | 1 | 0 | 0 | 1 | 1 | 1 | 8 |
| Rampinini et al. [234] | 1 | 1 | 1 | 1 | 1 | 1 | 1 | 0 | 1 | 1 | 1 | 10 |
| Voutselas et al. [235] | 1 | 1 | 1 | 1 | 1 | 1 | 1 | 0 | 1 | 1 | 1 | 10 |
| Kalapotharakos et al. [236] | 1 | 1 | 1 | 1 | 1 | 1 | 0 | 0 | 1 | 1 | 1 | 9 |
| Ostojic [237] | 1 | 1 | 1 | 1 | 1 | 1 | 1 | 0 | 1 | 1 | 1 | 10 |
| Arnason et al. [19] | 1 | 1 | 1 | 1 | 1 | 1 | 1 | 0 | 1 | 1 | 1 | 10 |
| Wisløff et al. [9] | 1 | 1 | 1 | 1 | 1 | 1 | 1 | 0 | 1 | 1 | 1 | 10 |
| Kraemer et al. [238] | 1 | 1 | 1 | 1 | 1 | 1 | 0 | 0 | 1 | 1 | 1 | 9 |
| Chamari et al. [239] | 1 | 1 | 1 | 1 | 1 | 1 | 1 | 0 | 1 | 1 | 1 | 10 |
| Askling et al. [240] | 1 | 1 | 1 | 1 | 1 | 1 | 0 | 0 | 1 | 1 | 1 | 9 |
| Ozcakar [241] | 1 | 0 | 1 | 1 | 1 | 1 | 1 | 0 | 1 | 1 | 1 | 9 |
| Helgerud et al. [242] | 1 | 1 | 1 | 1 | 1 | 1 | 0 | 0 | 1 | 1 | 1 | 9 |
| Casajús [243] | 1 | 0 | 1 | 1 | 1 | 1 | 0 | 0 | 1 | 1 | 0 | 7 |
| Cometti et al. [244] | 1 | 1 | 1 | 1 | 1 | 1 | 1 | 0 | 1 | 1 | 1 | 10 |
| Al-Hazzaa et al. [245] | 1 | 0 | 1 | 1 | 1 | 1 | 1 | 0 | 1 | 1 | 1 | 9 |
| Gür et al. [246] | 1 | 1 | 1 | 1 | 1 | 1 | 0 | 0 | 1 | 1 | 1 | 9 |
| Wisløff et al. [247] | 1 | 1 | 1 | 1 | 1 | 1 | 0 | 0 | 1 | 1 | 1 | 9 |
| Aagaard et al. [248] | 1 | 1 | 1 | 1 | 1 | 1 | 0 | 0 | 1 | 1 | 1 | 9 |
| Chin et al. [249] | 1 | 1 | 1 | 1 | 1 | 1 | 0 | 0 | 0 | 0 | 1 | 7 |
| Mangine et al. [250] | 1 | 0 | 0 | 0 | 1 | 1 | 1 | 0 | 1 | 0 | 0 | 5 |
| Poulmedis [251] | 1 | 0 | 0 | 0 | 1 | 1 | 0 | 0 | 0 | 0 | 1 | 4 |
| Rhodes et al. [252] | 1 | 1 | 1 | 1 | 1 | 1 | 0 | 0 | 1 | 0 | 1 | 8 |

**Supplementary Table S3** Characteristics of the studies included in the review

| **Author** | **Sample size** | **Playing standard** | **Age (years)** | **Study design** | **Country (championship)** |
| --- | --- | --- | --- | --- | --- |
| Maestroni et al. [66] | 55 | Senior professionals | 24.2 ± 3.1 | Repeated measures | Qatar (First Division) |
| Espada et al. [84] | 19 | Senior professionals | 23.2 ± 3.1 | Cross-sectional | Portugal (First Division) |
| Cossich et al. [85] | 46 | Senior professionals | 26.0 ± 6.0 | Cross-sectional | Brazil (First Division) |
| Byrkjedal et al. [86] | 16 | Senior professionals | 23.9 ± 4.3 | Intervention | Norway (Second Division) |
| Bishop et al. [64] | 19 | Elite youth | 17.6 ± 0.6 | Repeated measures | England (Premier League – Category 1 academy) |
| Wezenbeek et al. [87] | 84 | Senior professionals | 24.7 ± 4.6 | Repeated measures | Belgium |
| Guerra et al. [88] | 24 | Senior professionals | 23.0 ± 3.9 | Intervention | Brazil (First Division) |
| Papadakis et al. [89] | 44 | Senior professionals & elite youth | Senior professionals’s group: 24.6 ± 4.7  Elite youth group: 17.8 ± 4.7 | Cross-sectional | Greece (First Division) |
| Lahti et al. [90] | 161 | Senior professionals | 24.6 ± 5.4 | Repeated measures | France (Ligue 1) & Finland (Premier Finnish Division) |
| Bishop et al. [253] | 14 | Senior professionals | 27.5 ± 4.4 | Reliability study | England |
| Bongiovanni et al. [91] | 21 | Senior professionals | 27.2 ± 5.1 | Cross-sectional | Italy (Serie A) |
| Papla et al. [92] | 24 | Senior professionals | 24.8 ± 8.2 | Cross-sectional | Poland (First Division) |
| Boraczyński et al. [93] | 25 | Senior professionals | 18.4 to 29.7 | Intervention | Poland (First Division) |
| Misjuk and Rannama [94] | 28 | Senior professionals | 23.4 ± 4.5 | Cross-sectional | Estonia (Premium League) |
| Cadu et al. [95] | 23 | Senior professionals | 25.6 ± 3.5 | Repeated measures | France (Ligue 1) |
| Keiner et al. [15] | 48 | Elite youth | 17.5 ± 0.5 | Intervention | Germany (Bundesliga Elite Youth academy) |
| Schons et al. [96] | 48 | Senior professionals | 22.8 ± 3.4 | Cross-sectional | Brazil (Professional competition of the state of Rio Grande do Sul) |
| Freitas et al. [97] | 15 | Elite youth | 18.7 ± 0.5 | Repeated measures | Brazil (First Division) |
| Arregui-Martin et al. [98] | 38 | Elite youth | 18.7 ± 1.1 | Repeated measures | Spain (First Division) |
| Leporace et al. [99] | 24 | Senior professionals | 24.4 ± 4.6 | Cross-sectional | Brazil (First Division) |
| Querido and Clemente [100] | 18 | Elite youth | 18.5 ± 0.4 | Repeated measures | Portugal (U19 First Division) |
| Shalaj et al. [101] | 143 | Senior professionals | 24.1 ± 3.6 | Cross-sectional | Cosovo (First Division) |
| Manoel et al. [102] | 189 | Senior professionals | 26.2 ± 4.4 | Cross-sectional | Brazil (First Division) |
| Krespi et al. [103] | 158 | Elite youth | 17.1 ± 0.79 | Intervention | Croatia |
| Stern et al. [14] | 23 | Elite youth | 17.6 ± 1.2 | Intervention | England |
| Moreno-Pérez et al. [104] | 20 | Elite youth | 19.5 ± 1.6 | Cross-sectional | Spain (Second Division) |
| Correia et al. [105] | 24 | Senior professionals | 24.5 ± 4.2 | Cross-sectional | Portugal (First & Second Division) |
| Ribeiro-Alvares et al. [106] | 101 | Senior professionals & elite youth | Professional group: 21.0 ± 3.0  U20 group: 18.0 ± 1.0 | Cross-sectional | Brazil (State Premier League) |
| Suarez-Arrones et al. [107] | 50 | Elite youth | 18.8 ± 0.8 | Intervention | Spain (First Division – Reserve squad) |
| Van Klij et al. [108] | 217 | Senior professionals | 23 ± 3.8 | Repeated measures | Netherlands (1^st^ and 2^nd^ Division) |
| Ribeiro et al. [109] | 16 | Elite youth | 18.5 ± 0.5 | Intervention | Portugal |
| Lockie et al. [110] | 18 | Elite youth | 20.4 ± 1.5 | Cross-sectional | USA (Collegiate Division I) |
| Ishøi et al. [111] | 47 | Senior professionals & elite youth | Senior group: 20.8 ± 3.0  U19 group: 17.6 ± 1.0 | Cross-sectional | Denmark (First Division) |
| Bishop et al. [77] | 18 | Elite youth | 19.0 ± 2.2 | Repeated measures | England |
| Moreno-Pérez et al. [112] | 20 | Senior professionals | 20.9 ± 2.5 | Reliability study | Spain (Second Division) |
| Dolci et al. [113] | 11 | Elite youth | 18.5 ± 1.4 | Cross-sectional | Australia |
| Izovska et al. [43] | 40 | Senior professionals | 20.7 ± 1.6 | Cross-sectional | Czech Republic (First Division) |
| Nielsen et al. [114] | 37 | Senior professionals & elite youth | Senior group: 19.5 ± 1.8  U19 group: 17.2 ± 0.8 | Cross-sectional | Denmark (First Division) |
| Silva et al. [115] | 25 | Senior professionals | 28.1 ± 4.6 | Repeated measures | Qatar |
| Light et al. [116] | 47 | Senior professionals & elite youth | Senior group: 20.8 ± 3.0  U19 group: 17.6 ± 1.0 | Cross-sectional | Denmark (First Division) |
| Saccà et al. [117] | 74 | Elite youth | 16 to 20 | Repeated measures | Italy (Primavera Championship) |
| Enes et al. [118] | 24 | Senior professionals | 26.7 ± 3.9 | Cross-sectional | Brazil (First Division) |
| Ben Brahim et al. [119] | 34 | Elite youth | 18.8 ± 0.8 | Intervention | Tunisia (National U19 team) |
| Bishop et al. [63] | 35 | Elite youth | U23 group: 19.8 ± 1.1  U18 group: 17.5 ± 0.5 | Cross-sectional | England (Premier League academy) |
| Rodrigues Júnior et al. [120] | 20 | Senior professionals | 26.6 ± 4.0 | Repeated measures | Brazil (First Division) |
| Capaverde et al. [121] | 311 | Senior professionals & elite youth | 22.9 ± 5.6 (16 to 39) | Cross-sectional | Brazil (First and Second Division – 1^st^ team and U20) |
| Ribeiro-Alvares et al. [122] | 210 | Senior professionals | 24.2 ± 5.4 | Cross-sectional | Brazil (First and Second Division) |
| Scoz et al. [123] | 570 | Senior professionals | 17 to 36 | Cross-sectional | Brazil (First and Second Division) |
| Bourne et al. [124] | 204 | Senior professionals | 24.5 ± 5.1 | Cross-sectional | Australia (A-League) and England (Championship) |
| Ocarino et al. [125] | 134 | Senior professionals | 19.4 ± 2.6 | Cross-sectional | Brazil |
| Read et al. [126] | 203 | Senior professionals | 24.4 ± 4.7 | Cross-sectional | Qatar (First Division) |
| Beato et al. [18] | 143 | Senior professionals & elite youth | Professional group: 24 ± 5  Elite youth group: 18 ± 2 | Cross-sectional |  |
| Cardoso et al. [127] | 47 | Senior professionals | 17 to 34 | Cross-sectional | Germany (Bundesliga) |
| Loturco et al. [128] | 91 | Senior professionals & elite youth | Senior group: 25.2 ± 4.3  U20 group: 19.1 ± 0.4 | Cross-sectional | Brazil (First Division) |
| Papla et al. [129] | 15 | Senior professionals | 21.7 ± 0.7 | Cross-sectional | Poland (Second Division) |
| Loturco et al. [130] | 23 | Elite youth | 18.3 ± 0.7 | Intervention | Brazil (U20 team of a professional club) |
| Grazioli et al. [131] | 23 | Senior professionals | 26.3 ± 5.6 | Repeated measures | Brazil |
| Śliwowski et al. [132] | 100 | Senior professionals | 27.6 ± 4.9 | Cross-sectional | Poland (First Division) |
| Papadakis et al. [65] | 16 | Senior professionals | 25.6 ± 3.2 | Repeated measures | Cyprus (First Division) |
| Eustace et al. [133] | 34 | Senior professionals & elite youth | Senior group: 25.1 ± 3.8  Elite youth group: 17 ± 0.6 | Cross-sectional | England (Football League Division 2) |
| Hoppe et al. [17] | 54 | Elite youth | U21 group: 19.9 ± 0.3  U19 group: 17.6 ± 0.2 | Cross-sectional | Germany (Bundesliga Elite Youth academies) |
| Arcos et al. [134] | 46 | Senior professionals & elite youth | Senior group: 22.1 ± 2.0  U19 group: 18.3 ± 0.7  U17 group: 17.1 ± 0.3 | Cross-sectional | Spain (First Division) |
| Boraczyński et al. [135] | 25 | Senior professionals | 25.1 ± 4.5 | Cross-sectional | Poland (First Division) |
| Bianchi et al. [136] | 21 | Elite youth | 17.0 ± 0.8 | Intervention | Switzerland |
| Michaelides et al. [137] | 132 | Senior professionals | 25.2 ± 4.4 | Cross-sectional | Cyprus (First and Second Division) |
| Saidi et al. [138] | 18 | Senior professionals | 20.1±0.4 | Repeated measures | Tunisia (First Division) |
| Loturco et al. [139] | 49 | Senior professionals | 24.3 ± 4.2 | Cross-sectional |  |
| Northeast et al. [59] | 26 | Senior professionals | 25 ± 4 | Cross-sectional | England (Premier League) |
| Van Dyk et al. [140] | 41 | Senior professionals | 25 ± 4 | Repeated measures | Qatar |
| Moreno-Pérez et al. [141] | 71 | Senior professionals & elite youth | Senior group: 24.8 ± 4.2  U19 group: 17.4 ± 0.6 | Cross-sectional | Portugal (First Division) |
| López-Valenciano et al. [142] | 88 | Senior professionals | 25.5 ± 5.0 | Cross-sectional | Spain |
| Rago et al. [143] | 14 | Senior professionals | 27.6 ± 3.0 | Cross-sectional | Italy (Serie B) |
| Loturco et al. [144] | 25 | Elite youth | 17.6 ± 0.8 | Cross-sectional | Brazil |
| Mosler et al. [145] | 426 | Senior professionals | 25.2 ± 4.9 | Cross-sectional | Qatar (First Division) |
| Mosler et al. [146] | 438 | Senior professionals | 26 ± 6 | Cross-sectional | Qatar (First Division) |
| Los Arcos et al. [147] | 97 | Elite youth | 20.5 ± 1.5 | Repeated measures | Spain (First Division) |
| Almeida et al. [148] | 40 | Senior professionals | 20.7 (18-34) | Repeated measures | Brazil |
| Coratella et al. [149] | 27 | Elite youth | 18 to 21 | Cross-sectional | Italy (Serie A) |
| Suarez-Arrones et al. [150] | 14 | Elite youth | 17.5 ± 0.8 | Repeated measures | Italy (Serie A) |
| Enright et al. [80] | 19 | Elite youth | 18.3 ± 0.2 | Reliability study | England (Premier League) |
| Gil et al. [151] | 18 | Senior professionals | 22.4 ± 3.2 | Intervention | Brazil |
| Śliwowski et al. [152] | 31 | Elite youth | 18.6 ± 1.2 | Cross-sectional | Poland (First Division) |
| Van Dyk et al. [153] | 529 | Senior professionals | 25.9 ± 5 | Repeated measures | Qatar (First Division) |
| Buśko et al. [154] | 31 | Senior professionals | 20.9 ± 2.3 | Cross-sectional | Poland |
| Bakken et al. [155] | 369 | Senior professionals | 26.0 ± 4.7 | Cross-sectional | Qatar (First Division) |
| Lee et al. [156] | 25 | Senior professionals | 28.2 ± 3.4 | Reliability study | Hong Kong |
| Haugen [157] | 44 | Senior professionals |  | Repeated measures | Norway (First Division) |
| Murtagh et al. [158] | 23 | Elite youth | 18.1 ± 1.0 | Cross-sectional | England (Premier League Academy) |
| Van Dyk et al. [42] | 592 | Senior professionals | 25.8 ± 4.8 | Repeated measures | Qatar (First Division) |
| Kobal et al. [159] | 27 | Elite youth | 18.9 ± 0.6 | Intervention | Brazil (First Division) |
| Otero-Esquina et al. [13] | 36 | Elite youth | 17.0 ± 1.0 | Intervention | Spain (First Division) |
| Śliwowski et al. [160] | 111 | Senior professionals | 26 ± 5 | Cross-sectional | Poland (First Division) |
| Abade et al. [161] | 22 | Elite youth | 18.3 ± 0.5 | Intervention | Portugal (First Division) |
| Requena et al. [162] | 19 | Senior professionals | 26.2 ± 2.8 | Repeated measures | Spain (First Division) |
| Light and Thorborg [163] | 21 | Senior professionals | 21.3 ± 5 | Reliability study | England |
| Loturco et al. [164] | 27 | Elite youth | 18.4 ± 1.2 | Intervention | Brazil (First Division) |
| Mosler et al. [165] | 394 | Senior professionals | 26 ± 4.8 | Cross-sectional | Qatar (First Division) |
| Pareja-Blanco et al. [166] | 16 | Senior professionals | 23.8 ± 3.5 | Intervention | Spain |
| Krommes et al. [167] | 19 | Senior professionals | 24.0 ± 4.4 | Intervention | Denmark (Second Division) |
| Yanci and Los Arcos [168] | 20 | Elite youth | 20.6 ± 1.8 | Repeated measures | Spain (First Division) |
| Timmins et al. [45] | 152 | Senior professionals | 24.8 ± 5.1 | Cross-sectional | Australia |
| Belhaj et al. [169] | 21 | Senior professionals | 23.6 ± 3.4 | Cross-sectional | Morocco (First Division) |
| Van Dyk et al. [170] | 614 | Senior professionals | 24.7 ± 4.7 | Repeated measures | Qatar (First Division) |
| De Hoyo et al. [12] | 32 | Elite youth | 17.6 ± 1 | Intervention | Spain (First Division) |
| Fessi et al. [171] | 17 | Senior professionals | 23.7 ± 3.2 | Repeated measures | Qatar (First Division) |
| Kobal et al. [16] | 45 | Senior professionals & elite youth | Senior group: 22 ± 2.9  U20 group: 19 ± 0.6 | Cross-sectional | Brazil (Sao Paulo State First Division) |
| Spineti et al. [172] | 22 | Elite youth | 18.4 ± 0.4 | Intervention | Brazil (First Division) |
| Carvalho et al. [173] | 159 | Senior professionals | 25.7 ± 5 | Cross-sectional | Portugal (First and Second Division) |
| Rey et al. [174] | 62 | Senior professionals | 26.9 ± 4.9 | Cross-sectional | Spain |
| Martinez-Santos et al. [62] | 235 | Elite youth | 20.6 ± 1.8 | Repeated measures | Spain (First Division) |
| Loturco et al. [175] | 22 | Senior professionals | 23.8 ± 4.2 | Repeated measures | Brazil (Sao Paulo State First Division) |
| Noon et al. [176] | 14 | Elite youth | 17 ± 1 | Repeated measures | England (Category 2 Academy) |
| Enright et al. [177] | 15 | Elite youth | 17.3 ± 1.6 | Intervention | England (Premier League) |
| Ruas et al. [178] | 113 | Senior professionals | 25.2 ± 5.3 | Cross-sectional | Brazil (First Division) |
| Loturco et al. [75] | 23 | Senior professionals | 23.7 ± 4.4 | Intervention | Brazil (Sao Paulo State First Division) |
| Tsiokanos et al. [179] | 276 | Senior professionals | 26.4 ± 3.4 | Cross-sectional | Greece (First Division) |
| Pareja-Blanco et al. [180] | 21 | Senior professionals | 24.3 ± 4.6 | Cross-sectional | Morocco |
| Bogdanis and Kalapotharakos [181] | 18 | Senior professionals | 24.2 ± 1.1 | Cross-sectional | Greece (First Division) |
| Gil et al. [182] | 20 | Senior professionals | 23.3 ± 4.8 | Cross-sectional | Brazil |
| Loturco et al. [183] | 24 | Elite youth | 18.6 ± 0.6 | Intervention | Brazil (U20 Elite Championship) |
| Ardern et al. [184] | 42 | Senior professionals | 24.9 ± 5.3 | Cross-sectional | Australia |
| Loturco et al. [185] | 24 | Elite youth | 18.4 ± 0.7 | Intervention | Brazil (Sao Paulo State First Division) |
| Ruas et al. [186] | 102 | Senior professionals | 26 ± 5 | Cross-sectional | Brazil (South State First Division) |
| Arcos et al. [187] | 19 | Elite youth | 20.2 ± 1.9 | Repeated measures | Spain (First Division) |
| Booysen et al. [188] | 23 | Senior professionals | 23.0 ± 3.08 | Cross-sectional | South Africa (Second Division) |
| Owen et al. [189] | 10 | Senior professionals | 26.2 ± 4.3 | Repeated measures | Europe (UEFA Champions League) |
| Tol et al. [190] | 52 | Senior professionals | 24.9 (18-38) | Repeated measures | Qatar (First Division) |
| Edholm et al. [191] | 22 | Senior professionals | 25 (18-33) | Intervention | Sweden (First Division) |
| Brocherie et al. [192] | 16 | Senior professionals | 26.7 ± 4.0 | Cross-sectional | Qatar (National Team) |
| Haddad et al. [193] | 16 | Elite youth | 18.2 ± 1.2 | Intervention | Tunisia (First Division) |
| Arcos et al. [194] | 22 | Senior professionals | 19.9 ± 1.7 | Intervention | Spain (Second Division) |
| Koundourakis et al. [195] | 67 | Senior professionals | 24.7 ± 1.0 | Repeated measures | Greece (First and Second Division) |
| Koundourakis et al. [196] | 55 | Senior professionals | 25.1 ± 5.1 | Repeated measures | Greece (First Division) |
| Portella et al. [197] | 20 | Senior professionals | 26.3 ± 3.5 | Cross-sectional | Brazil (First Division) |
| Thorborg et al. [50] | 48 | Senior professionals | 23.7 ± 2.5 | Cross-sectional | Denmark (First to Fourth Division) |
| Requena et al. [198] | 25 | Senior professionals | 24 ± 5.8 | Cross-sectional | Spain (First Division) |
| Haugen et al. [199] | 939 | Senior professionals & elite youth | Senior group: 24.5 ± 3.8  Elite youth group: 18.5 ± 1.8 | Cross-sectional | Norway (National Team, First and Second Division) |
| Rebelo et al. [200] | 95 | Elite youth | 18.2 ± 0.6 | Cross-sectional | Portugal (First Division) |
| Menzel et al. [201] | 46 | Senior professionals | 24.8 ± 3.2 | Cross-sectional | Brazil (First Division) |
| Silva et al. [202] | 13 | Senior professionals | 25.7 ± 4.6 | Repeated measures | Portugal (First Division) |
| Loturco et al. [203] | 32 | Senior professionals | 19.1 ± 0.7 | Intervention | Brazil (State and National Championship) |
| Castagna and Castellini [204] | 35 | Elite youth | 20.3 ± 0.4 | Validity study | Italy (National Team) |
| Lago-Ballesteros [205] | 42 | Senior professionals | 25 ± 5.2 | Repeated measures | Spain (First Division) |
| Daneshjoo et al. [206] | 36 | Elite youth | 18.9 ± 1.4 | Intervention | Iran (First Division) |
| Boone et al. [207] | 289 | Senior professionals | 25.4 ± 4.9 | Cross-sectional | Belgium (First Division) |
| Chaouachi et al. [208] | 23 | Elite youth | 19 ± 1 | Cross-sectional | Tunisia (First Division) |
| Daneshjoo et al. [209] | 36 | Elite youth | 18.9 ± 1.0 | Intervention | Iran (First Division) |
| Whiteley et al. [210] | 216 | Senior professionals |  | Cross-sectional | Qatar (First Division) |
| Greco et al. [211] | 39 | Senior professionals | 24.2 ± 3.5 | Cross-sectional | Brazil |
| Requena et al. [212] | 14 | Senior professionals | 20.0 ± 3.6 | Intervention |  |
| Silva et al. [213] | 23 | Senior professionals | 25.7 ± 4.6 | Repeated measures | Portugal (First Division) |
| Jovanovic et al. [214] | 100 | Elite youth | 19 | Intervention | Croatia (First Division) |
| Helgerud et al. [215] | 21 | Senior professionals | 25 (20 to 31) | Intervention | Europe (UEFA Champions’ League) |
| Rønnestad et al. [216] | 19 | Senior professionals | 22 ± 2 | Intervention | Norway (Second Division) |
| Bogdanis et al. [217] | 22 | Senior professionals | 22.3 ± 1.1 | Intervention | Greece |
| Faude et al. [218] | 15 | Senior professionals & elite youth | 19.5 ± 3.0 | Repeated measures | Germany (Third Division and Highest U19 National League) |
| Zebis et al. [219] | 12 | Senior professionals | 24.5 ± 2.3 | Cross-sectional | Denmark (First and Second Division) |
| Cotte and Chatard [220] | 14 | Senior professionals |  | Cross-sectional | England (Premier League) |
| Henderson et al. [221] | 36 | Senior professionals | 22.6 ± 5.2 | Cross-sectional | England (Premier League) |
| Wong et al. [222] | 39 | Senior professionals | 22.8 ± 1.3 | Intervention | Hong Kong (First Division) |
| López-Segovia et al. [223] | 37 | Elite youth | 18.2 ± 0.7 | Repeated measures | Spain (First Division) |
| Till and Cooke [224] | 12 | Elite youth | 18.3 ± 0.7 | Intervention | England |
| Mujika et al. [225] | 20 | Elite youth | 18.3 ± 0.6 | Intervention | Spain (First Division) |
| Mujika et al. [226] | 34 | Senior professionals & elite youth | Senior group: 23.8 ± 3.4  Junior group: 18.4 ± 0.9 | Cross-sectional | Spain (First Division) |
| Sporis et al. [227] | 270 | Senior professionals | 28.3 ± 5.9 | Cross-sectional | Croatia (First Division) |
| Bravo et al. [228] | 22 | Elite youth | 17.3 ± 0.6 | Intervention |  |
| Croisier et al. [20] | 462 | Senior professionals | 26 ± 6 | Cross-sectional | Belgium, Brazil & France |
| Lehance et al. [21] | 39 | Senior professionals & elite youth | Professional group: 26.1 ± 3.5  U21 group: 19.5 ± 1.6 | Cross-sectional | Belgium (First Division) |
| Hoshikawa et al. [229] | 20 | Senior professionals | 22.6 ± 2.4 | Cross-sectional | Japan (First Division) |
| Clark et al. [230] | 42 | Senior professionals | 25 ± 3.5 | Repeated measures | England (Championship) |
| Ronnestad et al. [231] | 21 | Senior professionals | 23.5 ± 1.5 | Intervention | Norway (First Division) |
| Chamari et al. [232] | 15 | Elite youth |  | Cross-sectional | Tunisia (U23 National Olympic Team) |
| Cressey et al. [233] | 19 | Elite youth | 18 to 23 | Intervention | USA (Collegiate Division I) |
| Rampinini et al. [234] | 18 | Senior professionals | 26.2 ± 4.5 | Validity Study | Europe (UEFA Champions League) |
| Voutselas et al. [235] | 72 | Senior professionals | 25.1 ± 5.1 | Cross-sectional | Greece (First and Second Division) |
| Kalapotharakos et al. [236] | 54 | Senior professionals | 24.3 ± 3.7 | Cross-sectional | Greece (First Division) |
| Ostojic [237] | 30 | Senior professionals | 24 ± 2.5 | Cross-sectional | Serbia (First Division) |
| Arnason et al. [19] | 306 | Senior professionals | 24 (16 to 38) | Cross-sectional | Iceland (First and Second Division) |
| Wisløff et al. [9] | 17 | Senior professionals | 25.8 ± 2.9 | Cross-sectional | Norway (First Division) |
| Kraemer et al. [238] | 25 | Elite youth | 19.3 ± 0.9 | Repeated measures | USA (Collegiate Division I) |
| Chamari et al. [239] | 34 | Elite youth | 17.5 ± 1.1 | Cross-sectional | Tunisia (Junior National team & First Division) |
| Askling et al. [240] | 30 | Senior professionals | 25 ± 3.1 | Intervention | Sweeden (First Division) |
| Ozcakar [241] | 29 | Senior professionals | 23.6 ± 3.6 | Cross-sectional | Turkey (First Division) |
| Helgerud et al. [242] | 19 | Elite youth | 18.1 ± 0.8 | Intervention | Norway (First Division) |
| Casajús [243] | 15 | Senior professionals | 25.8 ± 3.2 | Repeated measures | Spain (First Division) |
| Cometti et al. [244] | 63 | Senior professionals | 24.6 ± 4.9 | Cross-sectional | France (Ligue 1 and Ligue 2) |
| Al-Hazzaa et al. [245] | 23 | Senior professionals | 25.2 ± 2.3 | Cross-sectional | Saudi Arabia (National Team) |
| Gür et al. [246] | 25 | Senior professionals & elite youth | 23.6 ± 3.6 | Cross-sectional | Turkey (First Division) |
| Wisløff et al. [247] | 29 | Senior professionals | 23.8 ± 3.8 | Cross-sectional | Norway (First Division) |
| Aagaard et al. [248] | 22 | Senior professionals | 22.8 ± 4.1 | Intervention | Denmark (First Division) |
| Chin et al. [249] | 24 | Senior professionals | 26.3 ± 4.2 | Cross-sectional | Hong Kong (First Division) |
| Mangine et al. [250] | 83 | Senior professionals & elite youth | 19 (16 to 26) | Cross-sectional | USA (National Team) |
| Poulmedis [251] | 18 | Senior professionals | 27.8 ± 3.4 | Cross-sectional | Greece |
| Rhodes et al. [252] | 16 | Elite youth | 20.1 ± 1.1 | Cross-sectional | Canada (National Olympic Team) |

**Supplementary Table S4** Reliability data for strength tests and outcome variables

| **Study** | **Test** | **Outcome variable** | **Reliability type** | **Reliability metric** | **Value** |
| --- | --- | --- | --- | --- | --- |
| Moreno-Pérez et al. [104] | Nordic hamstring testing | Peak force | Intraday | ICC | 0.977-0.999 |
|  |  |  |  | CV | 1.0-3.2 |
|  |  |  |  | SEM | 3.4-10.8 |
| Bishop et al. [253] | Nordic hamstring testing | Peak force | Interday | ICC | 0.54-0.73 |
|  |  |  |  | CV | 7.24 – 10.06 |
| Light and Thorborg [163] | Hip adductor isometric strength | Relative peak torque | Interday | ICC | 0.77-0.95 |
|  |  |  |  | SEM | 0.08-0.18 |
|  |  |  |  | SEM (%) | 3.2-7.8 |
|  |  |  |  | MDC (%) | 8.7-21.2 |
| Misjuk and Rannama [94] | Knee extensor isokinetic strength | Peak concentric torque 60 | Intraday | CV | 13 |
|  |  | Peak concentric torque 300 |  | CV | 14 |
|  |  | Conventional strength hamstrings/quadriceps ratio 60 |  | CV | 13 |
|  |  | Conventional strength hamstrings/quadriceps ratio 300 |  | CV | 12 |
|  |  | Relative peak concentric torque 60 |  | CV | 11 |
|  |  | Relative peak concentric torque 300 |  | CV | 11 |
|  | Knee flexor isokinetic strength | Peak concentric torque 60 |  | CV | 15 |
|  |  | Peak concentric torque 300 |  | CV | 20 |
|  |  | Relative peak concentric torque 60 |  | CV | 12 |
|  |  | Relative peak concentric torque 300 |  | CV | 15 |
| Keiner et al. [15] | Back squat | 1RM | Intraday | ICC | 0.97 |
| Ben Brahim et al. [119] | Half-back squat | 1RM | Intraday | ICC | 0.978 |
|  |  |  |  | CV | 3.1 |
|  |  |  |  | SEM | 1.71 |
| Eustace et al. [133] | Knee extensor isokinetic strength | Peak concentric torque 60 | Intraday | ICC | 0.87-0.91 |
|  |  | Peak concentric torque 180 |  | ICC | 0.87-0.89 |
|  |  | Peak concentric torque 270 |  | ICC | 0.83-0.84 |
|  |  | Functional strength hamstrings/quadriceps ratio 60 |  | ICC | 0.83-0.85 |
|  |  | Functional strength hamstrings/quadriceps ratio 180 |  | ICC | 0.80-0.83 |
|  |  | Functional strength hamstrings/quadriceps ratio 270 |  | ICC | 0.77-0.79 |
|  | Knee flexor isokinetic strength | Peak eccentric torque 60 |  | ICC | 0.84-0.88 |
|  |  | Peak eccentric torque 180 |  | ICC | 0.85-0.89 |
|  |  | Peak eccentric torque 270 |  | ICC | 0.82-0.83 |
| Boraczyński et al. [135] | Half-back squat | 1RM | Intraday | ICC | 0.94 |
|  | Knee extensor isometric strength | Peak torque |  | ICC | 0.88 |
| Coratella et al. [149] | Knee extensor isokinetic strength | Relative peak concentric torque 30 | Intraday | Cronbach's Alpha (α) | 0.961 |
|  |  | Relative peak concentric torque 300 |  | Cronbach's Alpha (α) | 0.937 |
|  |  | Relative peak eccentric torque 30 |  | Cronbach's Alpha (α) | 0.910 |
|  |  | Relative peak eccentric torque 300 |  | Cronbach's Alpha (α) | 0.906 |
|  | Knee flexor isokinetic strength | Relative peak concentric torque 30 |  | Cronbach's Alpha (α) | 0.942 |
|  |  | Relative peak concentric torque 300 |  | Cronbach's Alpha (α) | 0.899 |
|  |  | Relative peak eccentric torque 30 |  | Cronbach's Alpha (α) | 0.901 |
|  |  | Relative peak eccentric torque 300 |  | Cronbach's Alpha (α) | 0.910 |
| Enright et al. [80] | Half-back squat | 1RM | Interday | ICC | 0.99 |
|  |  |  |  | CV | 1.8 |
|  |  |  |  | SEM | 2.0 |
|  |  |  |  | MDC | 5.52 |
| Van Dyk et al. [42] | Knee extensor isokinetic strength | Peak concentric torque 60 | Interseason | ICC | 0.71 |
|  |  |  |  | SEM (%) | 24.5 |
|  |  | Peak concentric torque 60 |  | ICC | 0.79 |
|  |  |  |  | SEM (%) | 12.4 |
|  | Knee flexor isokinetic strength | Peak concentric torque 60  Peak concentric torque 300 |  | ICC | 0.63 |
|  |  |  |  | SEM (%) | 15.7 |
|  |  | Peak eccentric torque 60  Peak concentric torque 60 |  | ICC | 0.56 |
|  |  |  |  | SEM (%) | 14.5 |
|  |  | Peak concentric torque 300 |  | ICC | 0.52 |
|  |  |  |  | SEM (%) | 26.6 |
| Lee et al. [156] | Nordic hamstring testing | Break-point angle (°) | Interday | ICC | 0.94 |
|  |  |  |  | SEM | 3.44 |
|  |  |  |  | SEM (%) | 8.4 |
|  |  |  |  | MDC | 8.03 |
|  |  |  |  | MDC (%) | 19.7 |
| Spineti et al. [172] | Back squat ˄ | 1RM | Interday | ICC | 0.97 |
|  |  |  |  | SEM | 0.61 |
| Bogdanis et al. [217] | Half-back squat ˄ | 1RM | Intraday | ICC | 0.97 |
| López-Segovia et al. [223] | Isoinertial squat-loading test ˄ | Average mean concentric velocity | Intraday | ICC | 0.92-0.94 |

*ICC* intraclass correlation coefficient; *CV* coefficient of variation; *SEM* standard error of measurement; *MDC* minimal detectable change; *1RM* one repetition maximum

˄ Tests performed on Smith machine

**Supplementary Table S5** Reliability data for power tests and outcome variables

| **Study** | **Test** | **Outcome variable** | **Reliability type** | **Reliability metric** | **Value** |
| --- | --- | --- | --- | --- | --- |
| Maestroni et al. [66] | CMJ | Jump height | Intraday | CV | 2.7 |
|  |  |  |  | ICC | 0.978 |
|  |  |  |  | SEM | 1.4 |
|  |  | Relative peak power |  | CV | 2.1 |
|  |  |  |  | ICC | 0.966 |
|  |  |  |  | SEM | 1.4 |
|  |  | RSI mod |  | CV | 8.6 |
|  |  |  |  | ICC | 0.945 |
|  |  |  |  | SEM | 0.0 |
|  | Unilateral CMJ | Jump height |  | CV | 5.2-5.9 |
|  |  |  |  | ICC | 0.93-0.96 |
|  |  |  |  | SEM | 1.0 |
|  |  | Relative peak power |  | CV | 4.0-6.3 |
|  |  |  |  | ICC | 0.781-0.860 |
|  |  |  |  | SEM | 1.4-2.2 |
|  |  | RSI mod |  | CV | 8.0-10.8 |
|  |  |  |  | ICC | 0.893-0.907 |
|  |  |  |  | SEM | 0.0 |
| Krespi et al. [103] | CMJ | Jump height | Intraday | ICC | 0.927 |
|  |  |  |  | CV | 3.3 |
|  | SJ | Jump height |  | ICC | 0.91 |
|  |  |  |  | CV | 2.9 |
| Stern et al. [14] | CMJ | Jump height | Intraday | ICC | 0.95 |
|  |  |  |  | CV | 2.0 |
|  | Unilateral CMJ | Jump height |  | ICC | 0.95-0.96 |
|  |  |  |  | CV | 2.51-2.52 |
|  | DJ | RSI |  | ICC | 0.74 |
|  |  |  |  | CV | 7.69 |
|  | Unilateral DJ | RSI |  | ICC | 0.64-0.74 |
|  |  |  |  | CV | 6.27-6.38 |
|  | Standing broad jump test | Distance |  | ICC | 0.82 |
|  |  |  |  | CV | 2.99 |
|  | Single leg hop test | Distance |  | ICC | 0.78-0.88 |
|  |  |  |  | CV | 2.22-3.14 |
| Bishop et al. [64] | CMJ | Jump height | Intraday | ICC | 0.92 |
|  |  |  |  | CV | 3.17 |
|  |  | RSI mod |  | ICC | 0.95 |
|  |  |  |  | CV | 5.10 |
|  |  | Time to take-off |  | ICC | 0.96 |
|  |  |  |  | CV | 3.88 |
|  |  | Countermovement depth |  | ICC | 0.81 |
|  |  |  |  | CV | 8.64 |
|  | Unilateral CMJ | Jump height |  | ICC | 0.74-0.76 |
|  |  |  |  | CV | 4.26-4.61 |
|  |  | RSI mod |  | ICC | 0.90-0.93 |
|  |  |  |  | CV | 7.70-7.93 |
|  |  | Time to take-off |  | ICC | 0.96 |
|  |  |  |  | CV | 3.55-3.75 |
|  |  | Countermovement depth |  | ICC | 0.67-0.70 |
|  |  |  |  | CV | 8.64-8.66 |
| Misjuk and Rannama [94] | CMJ | Jump height | Intraday | CV | 15 |
| Keiner et al. [15] | SJ | Jump height | Intraday | ICC | 0.94 |
| Schons et al. [96] | CMJ | Jump height | Intraday | CV | 11.59 |
|  | SJ | Jump height |  | CV | 13.2 |
| Ribeiro et al. [109] | CMJ | Jump height | Intraday | ICC | 0.93 |
|  |  |  |  | CV | 3.5 |
|  | SJ | Jump height |  | ICC | 0.95 |
|  |  |  |  | CV | 3.6 |
| Bishop et al. [77] | Unilateral CMJ | Jump height | Intraday | ICC | 0.80-0.93 |
|  |  |  |  | CV | 7.90-9.63 |
|  |  | Concentric impulse |  | ICC | 0.75-0.88 |
|  |  |  |  | CV | 6.55-7.82 |
|  | Unilateral DJ | Jump height |  | ICC | 0.93-0.97 |
|  |  |  |  | CV | 6.38-7.30 |
|  |  | RSI |  | ICC | 0.88-0.96 |
|  |  |  |  | CV | 4.95-6.38 |
| Ben Brahim et al. [119] | CMJ | Jump height | Intraday | ICC | 0.99 |
|  |  |  |  | CV | 2.8 |
|  |  |  |  | SEM | 0.6 |
|  | SJ | Jump height |  | ICC | 0.99 |
|  |  |  |  | CV | 2.7 |
|  |  |  |  | SEM | 0.6 |
| Bishop et al. [63] | CMJ | Jump height | Intraday | ICC | 0.97 |
|  |  |  |  | CV | 2.47 |
|  |  |  |  | SEM | 0.69 |
|  | Unilateral CMJ | Jump height |  | ICC | 0.99 |
|  |  |  |  | CV | 1.98-2.02 |
|  |  |  |  | SEM | 0.33-0.35 |
| Boraczyński et al. [135] | VJ | Jump height | Intraday | ICC | 0.90 |
|  |  | Peak power |  | ICC | 0.92 |
| Coratella et al. [149] | CMJ | Jump height | Intraday | Cronbach's Alpha (α) | 0.875 |
|  | SJ | Jump height |  | Cronbach's Alpha (α) | 0.894 |
| Enright et al. [80] | CMJ | Jump height | Interday | ICC | 0.83 |
|  |  |  |  | CV | 4.3 |
|  |  |  |  | SEM | 1.69 |
|  |  |  |  | MDC | 4.7 |
|  | SJ | Jump height |  | ICC | 0.89 |
|  |  |  |  | CV | 3.7 |
|  |  |  |  | SEM | 1.41 |
|  |  |  |  | MDC | 3.9 |
| Gil et al. [151] | CMJ | Jump height | Intraday | ICC | 0.92 |
|  |  |  |  | CV | 2.15 |
|  |  |  |  | Pearson’s R | 0.87 |
|  | SJ | Jump height |  | ICC | 0.95 |
|  |  |  |  | CV | 2.12 |
|  |  |  |  | Pearson’s R | 0.91 |
|  | Jump squat | Mean power |  | ICC | 0.978 |
|  |  |  |  | CV | 3.75 |
|  |  |  |  | Pearson’s R | 0.95 |
|  |  | Mean propulsive power |  | ICC | 0.971 |
|  |  |  |  | CV | 3.30 |
|  |  |  |  | Pearson’s R | 0.94 |
| Los Arcos and Martins [147] | CMJ | Jump height | Interseason | CV | 5.0 |
| Haugen [157] | CMJ | Jump height | Intraday | ICC | 0.94 |
|  |  |  |  | CV | 3.1 |
| Otero-Esquina et al. [13] | CMJ | Jump height | Intraday | ICC | 0.99 |
|  |  |  |  | CV | 1.8 |
|  |  | Relative peak power |  | ICC | 0.95 |
|  |  |  |  | CV | 1.7 |
| Loturco et al. [164] | CMJ | Jump height | Intraday | ICC | 0.94 |
|  |  |  |  | CV | 3.5 |
|  | SJ | Jump height |  | ICC | 0.96 |
|  |  |  |  | CV | 3.1 |
| Pareja-Blanco et al. [166] | CMJ | Jump height | Intraday | ICC | 0.98 |
|  |  |  |  | CV | 3.1 |
| Kobal et al. [16] | CMJ | Jump height | Intraday | ICC | 0.96 |
|  | SJ | Jump height |  | ICC | 0.93 |
| Loturco et al. [175] | CMJ | Jump height | Intraday | ICC | 0.94 |
|  |  |  |  | CV | 3.5 |
|  | SJ | Jump height |  | ICC | 0.96 |
|  |  |  |  | CV | 3.1 |
| Gil et al. [182] | CMJ | Jump height | Intraday | ICC | 0.88 |
|  |  |  |  | CV | 3.96 |
|  | DJ |  |  | ICC | 0.975 |
|  |  |  |  | CV | 2.93 |
| Loturco et al. [183] | CMJ | Jump height | Intraday | ICC | 0.96 |
|  | Jump squat | Mean propulsive velocity |  | ICC | 0.94 |
| Loturco et al. [185] | CMJ | Jump height | Intraday | ICC | 0.95 |
|  | Standing broad jump test | Distance |  | ICC | 0.94 |
| Requena et al. [198] | VJ | Flight time | Interday | ICC | 0.99 |
|  |  |  |  | CV | 1.4 |
|  |  | Velocity at take-off |  | ICC | 0.97 |
|  |  |  |  | CV | 1.3 |
|  | DJ | Flight time |  | ICC | 0.95 |
|  |  |  |  | CV | 2.5 |
|  |  | Velocity at take-off |  | ICC | 0.90 |
|  |  |  |  | CV | 6.9 |
|  |  | Contact time |  | ICC | 0.97 |
|  |  |  |  | CV | 2.4 |
|  | Soccer-specific vertical jump | Flight time |  | ICC | 0.97 |
|  |  |  |  | CV | 2.5 |
|  |  | Velocity at take-off |  | ICC | 0.97 |
|  |  |  |  | CV | 2.4 |
| Rebelo et al. [200] | CMJ | Jump height | Intraday | Pearson’s r | 0.89 |
|  | SJ | Jump height |  | Pearson’s r | 0.97 |
| Daneshjoo et al. [206] | VJ | Jump height | Intraday | ICC | 0.98 |
| Boone et al. [207] | CMJ | Jump height | Intraday | ICC | 0.84 |
|  |  |  |  | Cronbach's Alpha (α) | 0.89 |
|  | SJ | Jump height |  | ICC | 0.87 |
|  |  |  |  | Cronbach's Alpha (α) | 0.90 |
| Silva et al. [213] | CMJ | Jump height | Intraday | ICC | 0.80-0.88 |
| Rønnestad et al. [216] | SJ | Jump height | Intraday | ICC | 0.97 |
| López-Segovia et al. [223] | CMJ | Jump height | Intraday | ICC | 0.95 |
|  |  |  |  | CV | 5.8 |
|  | CMJ (with external load on smith machine) |  |  | ICC | 0.93-0.97 |
|  |  |  |  | CV | 4-4.3 |
| Sporis et al. [227] | CMJ | Jump height | Intraday | ICC | 0.88 |
|  |  |  |  | Cronbach's Alpha (α) | 0.91 |
|  | SJ | Jump height |  | ICC | 0.75 |
|  |  |  |  | Cronbach's Alpha (α) | 0.73 |
| Chamari et al. [232] | Five jump test | Distance | Interday | ICC | 0.91 |
|  |  |  |  | CV | 2.2 |

*ICC* intraclass correlation coefficient; *CV* coefficient of variation; *SEM* standard error of measurement; *CMJ* countermovement jump; *RSI mod* reactive strength index modified; *SJ* squat jump; *DJ* drop jump; RSI reactive strength index; MDC minimal detectable change; *VJ* vertical jump with free arms
